# DeepFake electrocardiograms using generative adversarial networks are the beginning of the end for privacy issues in medicine

**DOI:** 10.1038/s41598-021-01295-2

**Published:** 2021-11-09

**Authors:** Vajira Thambawita, Jonas L. Isaksen, Steven A. Hicks, Jonas Ghouse, Gustav Ahlberg, Allan Linneberg, Niels Grarup, Christina Ellervik, Morten Salling Olesen, Torben Hansen, Claus Graff, Niels-Henrik Holstein-Rathlou, Inga Strümke, Hugo L. Hammer, Mary M. Maleckar, Pål Halvorsen, Michael A. Riegler, Jørgen K. Kanters

**Affiliations:** 1 SimulaMet, 0167 Oslo, Norway; 2 Oslo Metropolitan University, 0167 Oslo, Norway; 3 University of Copenhagen, 2200 Copenhagen N, Denmark; 4 Bispebjerg and Frederiksberg Hospital, 2400 Copenhagen NV, Denmark; 5 Aalborg University, Aalborg, Denmark; 6 Novo-Nordisk Foundation, Copenhagen, Denmark; 7 UiT The Arctic University of Norway, Tromsø, Norway

**Keywords:** Machine learning, Cardiovascular biology, Computational biology and bioinformatics

## Abstract

Recent global developments underscore the prominent role big data have in modern medical science. But privacy issues constitute a prevalent problem for collecting and sharing data between researchers. However, synthetic data generated to represent real data carrying similar information and distribution may alleviate the privacy issue. In this study, we present generative adversarial networks (GANs) capable of generating realistic synthetic DeepFake 10-s 12-lead electrocardiograms (ECGs). We have developed and compared two methods, named WaveGAN* and Pulse2Pulse. We trained the GANs with 7,233 real normal ECGs to produce 121,977 DeepFake normal ECGs. By verifying the ECGs using a commercial ECG interpretation program (MUSE 12SL, GE Healthcare), we demonstrate that the Pulse2Pulse GAN was superior to the WaveGAN* to produce realistic ECGs. ECG intervals and amplitudes were similar between the DeepFake and real ECGs. Although these synthetic ECGs mimic the dataset used for creation, the ECGs are not linked to any individuals and may thus be used freely. The synthetic dataset will be available as open access for researchers at OSF.io and the DeepFake generator available at the Python Package Index (PyPI) for generating synthetic ECGs. In conclusion, we were able to generate realistic synthetic ECGs using generative adversarial neural networks on normal ECGs from two population studies, thereby addressing the relevant privacy issues in medical datasets.

## Introduction

The use of artificial intelligence (AI) has increased in medicine over the past years. The goal of AI in medicine is to aid clinicians with decisions that are more accurate and to improve personalized medicine. The prominent prerequisite and foundation for AI is a large amount of high-quality clinical data.

With updates of the General Data Protection Regulation (GDPR) regulative in the EU, the free flow of data has been restricted to ensure patient consent and anonymity^1^. Even anonymized or de-identified data must not be shared between research groups in different countries, because combining few variables in an anonymized dataset, may allow for individual identification^2^. For example, knowing the zip code, birthday and sex is enough to identify 87% of US citizens^3^. The European GDPR rules are stricter than the US HIPAA rules for health data exchange^4^. EU demands that health data protection in a third country is essentially equivalent to that in the EU, which is not the case with the US HIPAA system^5^. All health data transfers require that informed consent is received from each patient, which makes most transatlantic collaboration impossible, if not planned in advance. However, large-scale, publicly available open-access medical datasets are required for personalized medicine to improve data-heavy machine learning solutions in medicine.

Generating realistic synthetic data is an alternative solution to the privacy issue. Synthetic data should contain all the desired characteristics of a specific population, but without any sensitive content, making it impossible to identify individuals. Therefore, properly generated synthetic data is a solution to the privacy problem which enables data sharing between research groups.

An electrocardiogram (ECG) is a voltage time series that reflects the electric currents within the heart. An ECG is a widely used, easy applicable and inexpensive clinical screening procedure to detect cardiac diseases. With the use of multiple electrodes, 3D propagation of cardiac electric impulses is obtained and plotted as a standard 10-s 12-lead ECG.

In this paper, we showcase synthetic ECGs as an example of complex medical data. Synthetic ECGs have been a topic of interest and research for many years. McSharry et al.^6^ and Sayadi et al.^7^ proposed mathematical dynamical models to generate continuous ECG signals, but these models were restricted to only one lead and did not reflect the distribution found in the normal population, nor did they give any insight in the mechanisms behind any disease.

Generative adversarial networks (GAN) were introduced in 2014 by Goodfellow et al.^8^ to generate synthetic data using multi-layer perceptrons. A GAN consists of two deep neural networks: a generator network, which creates signals (here ECGs) from random noise, and a discriminator network, which evaluates whether an ECG presented to it is real or fake. During training, a mix of real ECGs (from the underlying population) and generated DeepFake ECGs (from the generator) are presented to the discriminator, which assigns a score to the ECG (high score for real, low score for fake). As training proceeds, both the generator and the discriminator improve in performance until an equilibrium is reached^9^. Later, Radford et al.^10^ developed a convolutional GAN to generate synthetic images, which is well suited for images.

Since ECGs are time series data, our initial approach was to use a WaveGAN^11^ which is capable of generating sound signals. The classical WaveGAN is only able to output a single channel time series, so we modified the WaveGAN to generate 8 ECG channels (denoted WaveGAN*) instead of audio signals. We then introduced a novel DeepFake ECG U-net generative model, called Pulse2Pulse, which was inspired by the WaveGAN^11^, and we compared our Pulse2Pulse GAN to the WaveGAN*.

In this paper, we thus present two GANs with the ability to generate an unlimited number of 10-s 12-leads synthetic “DeepFake” ECGs as a solution to overcome the privacy issues related to real ECG data. These DeepFake ECGs can be openly distributed and freely downloaded as open access and used by other scientists to develop ECG algorithms.

## Results

**Figure 1:**
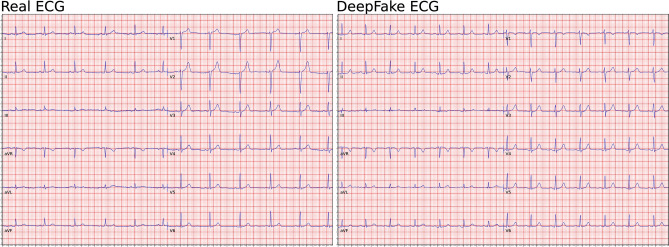
Comparison of examples of a real ECG (left lane) and a DeepFake ECG (right lane). See supplementary Figure S1 for 20 more randomly chosen pairs of real and DeepFake ECGs.

**Figure 2:**
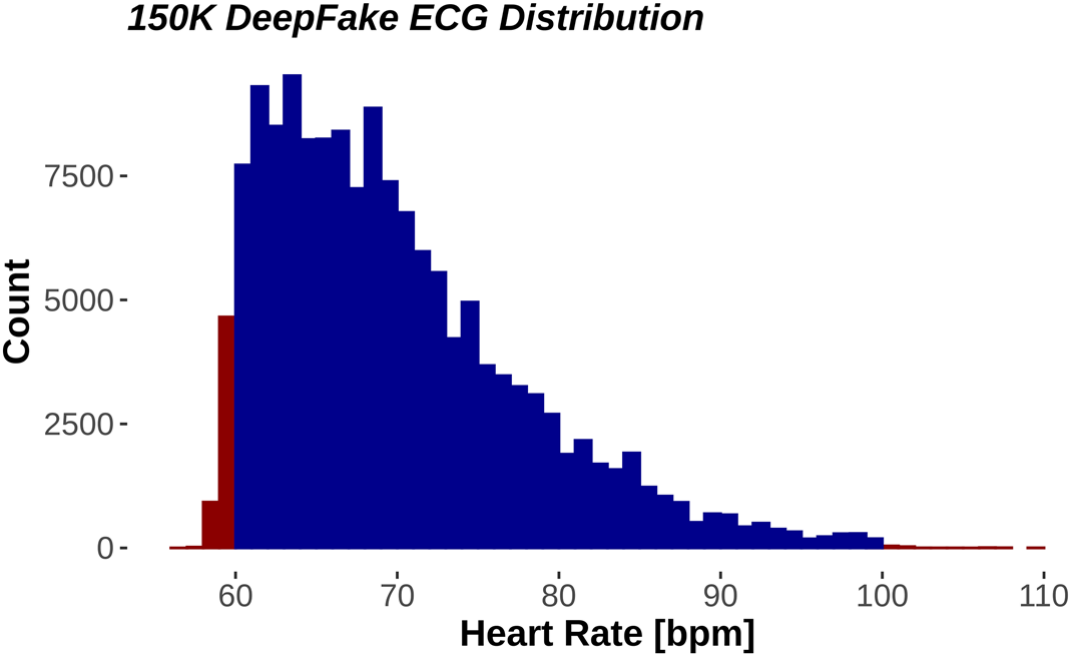
Distribution of heart rates in all 150,000 DeepFake electrocardiograms. Red fill denotes outside the normal heart rate range. Blue fill is within normal heart rate range (60–99).

**Figure 3:**
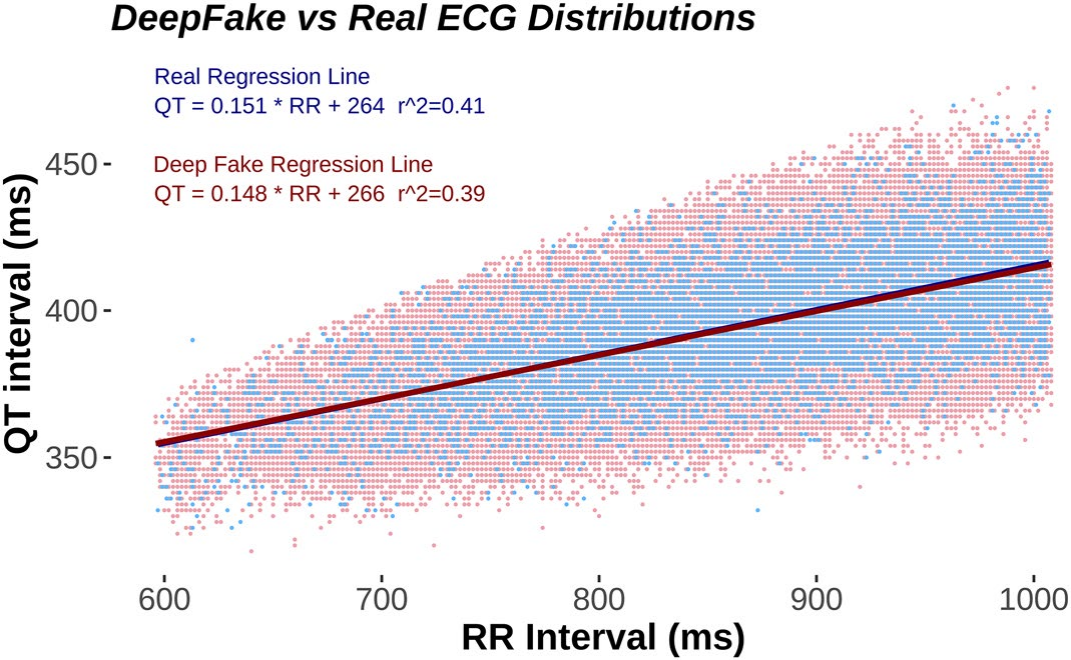
Scatter plot of the QT/RR interval relationship where Real ECG are shown in blue and normal DeepFakes in red. DeepFake dots are nudged 1 ms to the left for visibility. Note that there are 121,977 normal DeepFakes and only 7233 Real ECG making the DeepFake distribution more pronounced. As seen by the correlation coefficient r2, the real and the fake DeepFake ECGs are similarly distributed.

We used ECGs from two population studies (GESUS^12^ and Inter99^13^). To avoid chimeras between normal and abnormal ECGs, we only trained the neural network with ECGs classified as normal by the MUSE 12SL (version 2.43). As shown in Table 1, both the WaveGAN* and Pulse2Pulse improved during training expressed as the percentage of DeepFake ECGs classified by the commercial ECG interpretation program MUSE 12SL as normal ECGs. The Pulse2Pulse GAN trained faster than the WaveGAN* and had a better performance (expressed as fraction of ECGs classified as normal by the MUSE) compared to the WaveGAN* at their respective optimal number of training epochs (Table 1). Figure 1 shows a comparison of real and DeepFake ECGs, and the Supplementary Figure S1 shows twenty randomly chosen DeepFake ECGs. Figure 2 shows the distribution of heart rates in the DeepFakes. By clinical definition Normal ECGs heart rates are between 60 and 99 beats per minute. The MUSE 12SL^14^ classified 129 DeepFakes (0.5%) as sinus tachycardia (fast heart rate ≥ 100) and 2863 (10.2%) as sinus bradycardia (slow heart rate < 60). Figure 3 shows that the well-known established correlation between the QT interval and the RR interval^15^ was preserved. All covariance structures can be seen in the Supplementary Figure S2.

**Table 1:** Quantitative difference between WaveGAN* and Pulse2Pulse GAN in the initial training for determining the optimal network and optimal number of epochs.

Checkpoint (epochs)	Fraction of DeepFake ECGs classified as Normal (%)	
	WaveGAN*	Pulse2Pulse
500	20.9	78.7
1000	69.5	81.2
1500	71.2	78.8
2000	**72.5**	79.7
2500	71.3	**81.6**
3000	65.3	81.5

The best values are bolded for each GAN.

**Figure 4:**
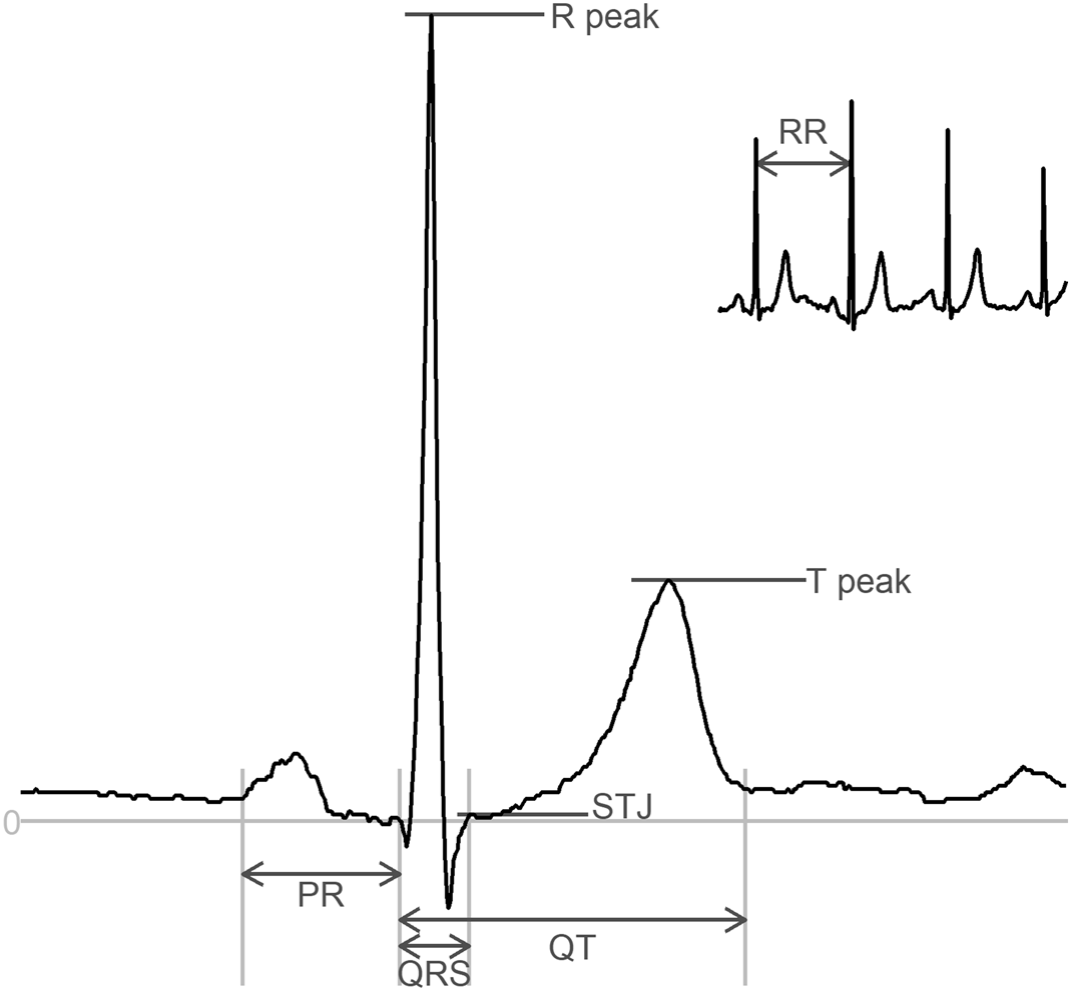
An ECG complex with the nomenclature of intervals (QT, QRS, P duration) and Amplitudes (STJ, R, T) and RR-interval (which can be converted to heart rate (HR) as HR = 60/RR interval.

The generated DeepFake ECGs can be downloaded at OSF.io (https://osf.io/6hved/) with the corresponding ground truth parameters for the QT, RR, PR and QRS intervals and the P, STJ, R, and T amplitudes (see Fig. 4 for ECG wave/interval naming terminology) delivered by the MUSE 12SL system. The DeepFake ECGs may be freely used for scientific use or commercial algorithm development if this paper is properly cited.

Using the Pulse2Pulse model from the optimal number of epochs (2500), we generated 150,000 DeepFake ECGs. To ensure that these ECGs were realistic, we uploaded the 150,000 ECGs to the GE MUSE system and analyzed them using the 12SL algorithm. We found that 81.3% of the 150,000 DeepFake ECGs were classified as “Normal ECG” (vs. 81.6% in the initial training). Table 2 compares real vs. DeepFake ECGs using eight ECG properties (heart rate, P duration, QT interval, QRS duration, PR interval, STJ amplitude, R amplitude, and T amplitude extracted using MUSE 12SL. See Fig. 4 for ECG nomenclature). The real data included all ECGs from GESUS and Inter99 classified as “Normal ECG” which were used for training. DeepFake ECGs are presented both as all 150,000 generated ECGs and the subset classified as Normal ECG. Supplementary Table S4 summaries the most common reasons for classifying DeepFake ECGs as Non-Normal ECGs.

**Table 2:** Mean, standard deviation (std), 2.5%, and 97.5% percentile for standard ECG parameters in real and fake ECGs.

		Real—normal (7233)				Pulse2Pulse—normal (121,977)				Pulse2Pulse—all (150,000)			
		Mean	Std	2.5%	97.5%	Mean	Std	2.5%	97.5%	Mean	Std	2.5%	97.5%
Heart rate	BPM	70	8	60	90	70	7	60	88	70	8	60	89
P duration	ms	105	12	82	130	117	17	86	152	118	17	84	152
QT interval	ms	395	21	352	436	395	20	354	436	395	22	352	436
QRS duration	ms	90	9	74	110	92	9	78	112	93	10	78	114
PR interval	ms	156	19	120	198	158	17	126	192	159	19	124	194
STJ amplitude (V5)	µV	2	27	− 44	58	18	33	− 44	87	16	36	− 54	87
R amplitude (V5)	µV	1287	402	600	2163	1275	367	620	2026	1273	402	566	2094
T amplitude (V5)	µV	343	137	126	664	366	135	156	668	361	141	141	673

*BPM* beats per minute.

## Discussion

Although deep learning has previously been used for ECG analysis^16,17^, this study is the first study to generate realistic synthetic 10-s 12-lead DeepFake ECGs. We demonstrate that the characteristics of the real ECGs were preserved with the DeepFake ECGs.

In our study, nearly one fifth of the DeepFake ECGs were not recognized as Normal ECGs (Non-Normal) by the commercial MUSE 12SL ECG analyzer (no ECGs were rejected as being invalid). Many ECG parameters use hard boundaries in distinguishing between Normal and Non-Normal. For example, a normal heart rate is by definition located between 60 to 99 beats per minute. Since we trained our model only on Normal ECGs, the input distribution for the GAN was a truncated asymmetric distribution. Thus, the clinically defined boundaries are skewed compared to the normal distribution of heart rates. The left truncation (at low heart rates) will discard more individuals than the right truncation (at high heart rates), and the final distribution of the real ECGs will be close to a truncated normal distribution with asymmetric truncations. The GAN will generally learn that heart rates outside 60–99 are not valid, but small deviations will occur as seen in Fig. 2 and Table 2. Since similar boundaries exist for many ECG parameters (for example PR interval > 220 ms or QRS Interval < 120 ms) sharp truncations occur with several ECG parameters. This could lead to the exclusion of some DeepFake ECGs, simply because the ECG intervals or amplitudes were marginally outside the normal range. Most ECG amplitudes and intervals were similar between real ECGs and DeepFake ECGs. It is noteworthy that the STJ amplitude and the P duration had the greatest deviation between real ECGs and DeepFake ECGs. This may be because both STJ and P amplitudes are small, and that the network may tend to focus on larger waves such as the R and T waves. Following this theory, the network would to some extent neglect the smaller waves and features thereby introducing a larger uncertainty. Future networks may improve the ECG generation using conditional GANs to give more attention to smaller signal features. The Pulse2Pulse model was able to preserve the covariance structure between different ECG features, as seen in the most important relationship the QT/RR relationship which is known to have prognostic importance^18^.

A challenging task is to define the optimal number of epochs for training. GANs tend to become unstable during the training process with the risk of the generator producing unrealistic output. To get an unbiased estimate on how well the trained GAN performs, we used the commercial MUSE 12SL system which automatically and reliably evaluates an ECG with a sensitivity of 99.9% and specificity of 100%^19^. Although the ECG discarded by the MUSE 12 SL may only have minimal abnormalities (like a heart rate of 59.9 bpm where 60 bpm is normal), the filtering of the DeepFake ECGs ensures that the best epoch is chosen without bias. It also ensures that the resulting ECGs are normal not only according to the discriminator, but also according to one of the most widely used ECG system in hospitals worldwide.

Personalized medicine depends on big data, which is frequently facilitated by international collaborations to ensure large datasets for both researchers and industry. However, privacy and general data protection regulation rules are major obstacles for sharing data between researchers from different institutions or countries or with the industry^20^.

In conclusion, by constructing synthetic signals from real patients which retain the same clinical information as was present in the real dataset, we have paved a new way to overcome privacy and ethical^21^ concerns for data sharing. The synthetic data generated by our Pulse2Pulse GAN are not linked to any specific patients but to the entire population, and therefore the ECGs prove useful for data scientists and the industry in developing novel algorithms for ECG analysis. The approach is not limited to ECGs but could be generalized to all medical multichannel data, e.g., electroencephalography and electromyography. Therefore, the DeepFake ECGs generated from the Pulse2Pulse model can be used as a replacement to overcome the privacy constraints in real medical datasets.

## Methods

GAN models were first introduced by Goodfellow et al.^8^. In a GAN, two deep neural networks termed the generator (G) and the discriminator (D) are combined to achieve the generation task. The main goal of the generator is to produce a data sample input [ECG(z)] from random noise (z) to present to the discriminator. The discriminator is tasked with differentiating between real and fake data, thus forcing the generator to improve performance. The generator and discriminator are trained together in a competition (minmax game). When a steady state is reached, the training halts and the generator will generate realistic synthetic ECGs.

### Data preparation

We used two combined datasets: the Danish General Suburban Population Study^12^ (GESUS) and the Inter99 study^13^ (CT00289237, ClinicalTrials.gov). GESUS consists of 8939 free-living subjects, and Inter99 consists of 6667 free-living subjects with an available digital ECG. To avoid generation of hybrid ECGs with mixed ECG abnormalities not occurring in real persons (e.g., to both be in sinus rhythm and atrial fibrillation at the same time which is impossible), we excluded ECGs who were not classified as normal (n = 8348) leaving 7233 Normal ECGs for training.

A 10-s 12-lead ECG consists only of 8 independent channels since 4 of the channels are simply trigonometric rotations of the two first channels. Therefore, the input ECG signal is 5000 × 8 data points (corresponding to 10 s with 500 samples per sec × 8 channels). We calculated the missing four channels with trigonometric functions to create the classic 12-channels ECG from 8-channels ECG.

#### WaveGAN*

**Figure 5:**
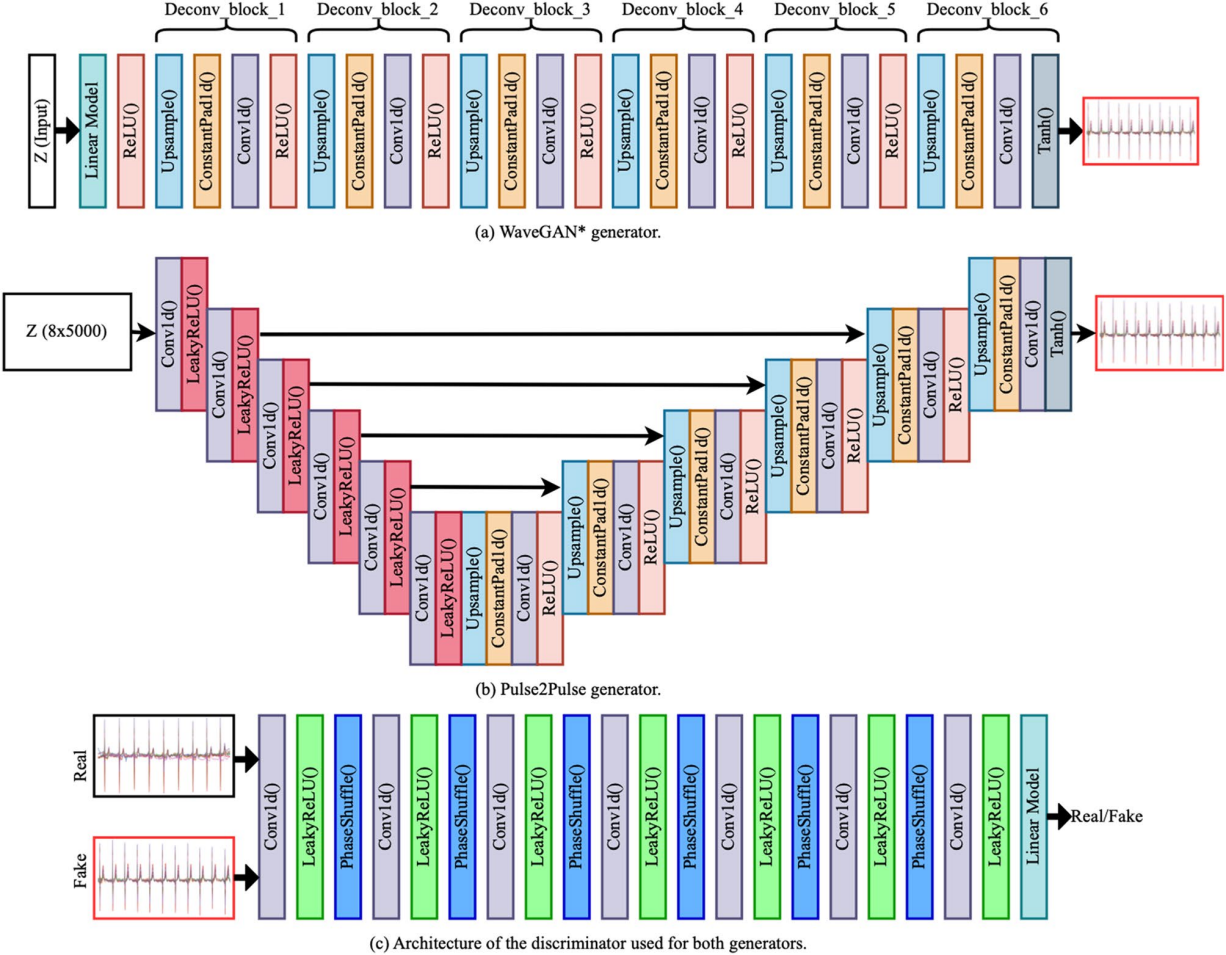
Model architectures of the generators and the discriminator used to generate synthetic ECGs. WaveGAN* uses a 1D noise vector with 100 points. Pulse2Pulse uses a 2D noise vector with size of 8 × 5000 as input, same as the output ECG size.

The input to WaveGAN* is a 1D 100 × 1 random noise vector sampled from the uniform distribution (mean = 0, std = 1) which passes through six deconvolution blocks to generate the desired output of 5000 × 8 samples (Fig. 5a). The deconvolution blocks were built from a series of four layers: an up-sampling layer, a constant padding layer, a 1D-convolution layer, and a ReLU activation function consecutively. This implementation is deeper than the original architecture, which uses five deconvolution blocks used to generate synthetic music samples. Table S1 has comprehensive details of our WaveGAN* generator network.

#### Pulse2Pulse

The implementation of the Pulse2Pulse architecture (Fig. 5) is inspired by the U-Net architecture^22^, which was used for image segmentation. However, our Pulse2Pulse implementation is different from the original U-net implementation because the Pulse2Pulse implementation uses 1D convolutional layers for ECG signal generation as opposed to the 2D convolutional layers used for the original image segmentation task. The Pulse2Pulse network takes an 8 × 5000 noise vector, i.e., the same dimension as the output ECG. The noise is passed through six down-sampling blocks followed by six up-sampling blocks as illustrated in Fig. 5b. Each down-sampling block consists of a 1D-convolution layer followed by a Leaky ReLU activation. The up-sampling block is similar to the deconvolution block used in WaveGAN*. In down-sampling, we have used Leaky ReLU instead of the ReLU layer used in the up-sampling to match the down-sampling operations to the discriminator. In addition to the up-sampling and down-sampling, the major modification is a bypass option, which concatenates the down-sampling block features with the up-sampling block features (represented by the black arrows in Fig. 5b). To facilitate for this concatenation, we doubled the input size of up-sampling blocks compared to WaveGAN* up-sampling blocks. More details about the Pulse2Pulse generator network are shown in the Supplementary Table S2.

#### Discriminator

The same discriminator was used by WaveGAN* and Pulse2Pulse to discriminate between real and fake ECGs (Fig. 5c). We used seven convolution layers (the original WaveGAN^11^ has five layers), and each convolution layer is followed by a Leaky ReLU activation and the phase shuffle layer introduced in the original WaveGAN paper^11^. The discriminator takes an ECG as input (5000 samples × 8 channels) and outputs a score how close the ECG are to be determined fake or real. Complete details about our discriminator network are given in the Supplementary Table S3.

#### Training

The models were trained on a Ubuntu workstation with two Xeon processors and a GeForce NVIDIA RTX 2080ti running the Pytorch deep learning framework^23^. We ran all our experiments (generators + discriminator) using the Adam^24^ optimizer with a learning rate of 0.0001, β1-value of 0.5, and β2-value of 0.9. As loss function, we used gradient clipping WGAN-GP^25^, to ensure faster and better convergence. Similar to the audio generation paper of WaveGAN^11^, we updated (backpropagated) the discriminator five times per update of the generator. We used a batch size of 32, which is half of the original batch size of 64 used in the original WaveGAN paper, because we used larger networks than the WaveGAN networks. We kept the training process until 3000 epochs (~ 10 days computing time) because we experienced unstable training curves for both WaveGAN* and Pulse2Pulse afterwards.

#### DeepFake ECGs

For evaluation of our two GAN models, we initially generated 10,000 ECGs from every 500 epochs until 3000 epochs from each GAN model. The DeepFake ECGs were transferred to the MUSE system and evaluated by the MUSE 12SL algorithm v. 2.43^14^, and we used the fraction of DeepFake ECGs described as Normal as the metric (because we only used Normal Real ECGs for the training). Using the best epoch for the best GAN, we generated 150,000 DeepFake ECGs. These DeepFakes were also evaluated by the MUSE 12SL.

## Supplementary Information

Supplementary Information.

## Data Availability

The Normal DeepFake ECGs are available at OSF (https://osf.io/6hved/) with corresponding MUSE 12SL ground truth values freely downloadable and usable for ECG algorithm development. The DeepFake generative model is available at https://pypi.org/project/deepfake-ecg/ to generate only synthetic ECGs.

## Code availability

The complete source code of all networks discussed in paper are available at GitHub (https://github.com/vlbthambawita/deepfake-ecg).
