# Supplementary material for: DeepFake electrocardiograms using generative adversarial networks are the beginning of the end for privacy issues in medicine
Source: Sci Rep. 2021 Nov 9;11:21896. doi: 10.1038/s41598-021-01295-2 (PMC8578227; doi:10.1038/s41598-021-01295-2)
Supplement: Supplementary file 1 — Supplementary Information. [file 41598_2021_1295_MOESM1_ESM.pdf]

DeepFake electrocardiograms using generative adversarial networks are the beginning of the end for privacy issues in medicine

Vajira Thambawita[1,2+], Jonas L. Isaksen[3+], Steven A. Hicks[1,2], Jonas Ghouse[3], GustavAhlberg[3], Allan Linneberg[3,4], Niels Grarup[3], Christina Ellervik[3], Morten Salling Olesen[3], Torben Hansen[3], Claus Graff[5], Niels-Henrik Holstein-Rathlou[6], Inga Strümke[1], Hugo L. Hammer[1,2], Mary M. Maleckar[1,2], Pål Halvorsen[1,2], Michael A. Riegler[1,7+*], and Jørgen K. Kanters[3+*]

[1]SimulaMet, Oslo 0167, Norway

[2]Oslo Metropolitan University, Oslo 0167, Norway

[3]University of Copenhagen, DK2200 Copenhagen N, Denmark

[4]Bispebjerg and Frederiksberg Hospital, DK2400 Copenhagen NV, Denmark

[5]Aalborg University, Aalborg, Denmark

[6]Novo-Nordisk Foundation, Copenhagen, Denmark

[7]UiT The Arctic University of Norway, Tromsø, Norway

*Corresponding Authors jkanters@sund.ku.dk, michael@simula.no, vajira@simula.no

+these authors contributed equally to this work

Supplementary materials

Table S1. Full details of the WaveGAN* Generator network. z = input noise vector, model dimension (d) = 50, batch size (n) = 32, channels (c) = 8.

**Operation**	**Kernel**	**Size**
Input z		(n,100)
Dense 1	(100,10d)	(n,10d)
Reshape		(n,5d,2)
ReLU		(n,5d,2)
Deconv_block_1 (stride=1,upsample=5)	(25,5d,5d)	(n,5d,10)
Deconv_block_2 (stride=1,upsample=5)	(25,5d,3d)	(n,3d,50)
Deconv_block_3 (stride=1,upsample=5)	(25,3d,d)	(n,d,250)
Deconv_block_4 (stride=1,upsample=2)	(25,d,d/2)	(n,d/2,500)
Deconv_block_5 (stride=1,upsample=5)	(25,d/2,d/5)	(n,d/5,2500)
Deconv_block_6 (stride=1,upsample=2)	(25,d/5,c)	(n,c,5000)

Table S2. Full details of the Generator network of Pulse2Pulse. z = noise vector (pseudo ECG), model dimension (d) = 50, batch size (n) = 32, channels (c) = 8. Deconvolution layers with * mark take output features from the 2nd, 3rd, 4th, and 5th convolutional layers and concatenate into channel dimension.

Operation	Kernel	Size
Input z		(n,c,5000)
Conv1D (Stride=2)	(25,c,d/5)	(n,d/5,2500)
LReLU (α=0.01)		(n,d/5,2500)
Conv1D (Stride=5)	(25,d/5,d/2)	(n,d/2,500)
LReLU (α=0.01)		(n,d/2,500)
Conv1D (Stride=2)	(25,d/2,d)	(n,d,250)
LReLU (α=0.01)		(n,d,250)
Conv1D (Stride=5)	(25,d,3d)	(n,3d,50)
LReLU (α=0.01)		(n,3d,50)
Conv1D (Stride=5)	(25,3d,5d)	(n,5d,10)
LReLU (α=0.01)		(n,5d,10)
Conv1D (Stride=5)	(25,5d,5d)	(n,5d,2)
LReLU (α=0.01)		(n,5d,2)
Deconv_block_1 (stride=1,upsample=5)	(25,5d,5d)	(n,5d,10)
Deconv_block_2* (stride=1,upsample=5)	(25,5dx2,3d)	(n,3d,50)
Deconv_block_3* (stride=1,upsample=5)	(25,3dx2,d)	(n,d,250)
Deconv_block_4* (stride=1,upsample=2)	(25,dx2,d/2)	(n,d/2,500)
Deconv_block_5* (stride=1,upsample=5)	(25,(d/2)x2,d/5)	(n,d/5,2500)
Deconv_block_6 (stride=1,upsample=2)	(25,d/5,c)	(n,c,5000)

Table S3. Details of the discriminator network. Model dimension (d) = 50, channels (c) = 8, batch size (n) = 32.

Operation	Kernel Size (width,channels,filters)	Output Shape
Input x or G(z)		(n,c,5000)
Conv1D (Stride=2)	(25,c,d)	(n,d,2499)
LReLU (α=0.2)		(n,d,2499)
Phase Shuffle (n=2)		(n,d,2499)
Conv1D (Stride=2)	(25,d,2d)	(n,2d,1249)
LReLU (α=0.2)		(n,2d,1249)
Phase Shuffle (n=2)		(n,2d,1249)
Conv1D (Stride=2)	(25,2d,5d)	(n,5d,624)
LReLU (α=0.2)		(n,5d,624)
Phase Shuffle (n=2)		(n,5d,624)
Conv1D (Stride=2)	(25,5d,10d)	(n,10d,311)
LReLU (α=0.2)		(n,10d,311)
Phase Shuffle (n=2)		(n,10d,311)
Conv1D (Stride=2)	(25,10d,20d)	(n,20d,78)
LReLU (α=0.2)		(n,20d,78)
Phase Shuffle (n=2)		(n,20d,78)
Conv1D (Stride=2)	(25,20d,25d)	(n,25d,19)
LReLU (α=0.2)		(n,25d,19)
Phase Shuffle (n=2)		(n,25d,19)
Conv1D (Stride=2)	(25,25d,100d)	(n,100d,5)
LReLU (α=0.2)		(n,100d,5)
Linear		(n,25000,1)

Table S4. MUSE 12SL diagnoses for the Non-Normal DeepFake ECGs. Note that an ECG may have several ECG diagnoses. The table shows only diagnoses with at least 250 occurrences.

**Total number of Non-Normal DeepFake ECGs**	**28023**
Non-Specific ST abnormality	7176
Sinus Bradycardia	2863
Inferior Infarct	2540
Left Ventricular Hypertrophy	1777
Non-Specific intraventricular delay	1610
Low Voltage QRS	1566
Fusion complexes	1244
Anterior Infarct	1238
ACUTE MI/STEMI	1003
Left Axis Deviation	826
Lateral Infarct	773
Premature Ventricular complexes	748
Septal Infarct	745
Undetermined Rhythm	630
Premature Atrial Complexes	615
With short PR	614
With 1 degree AV block	584
Non-specific T wave abnormality	526
Prolonged QT	460
Non-specific interventricular block	444
ST elevation (inferior injury)	405
Rightward axis	389
Aberrant conduction	384
Left Ventricular Hypertrophy	318
Moderate Left Ventricular Hypertrophy	310
Incomplete Right Bundle Branch Block	289
Left atrial enlargement	268

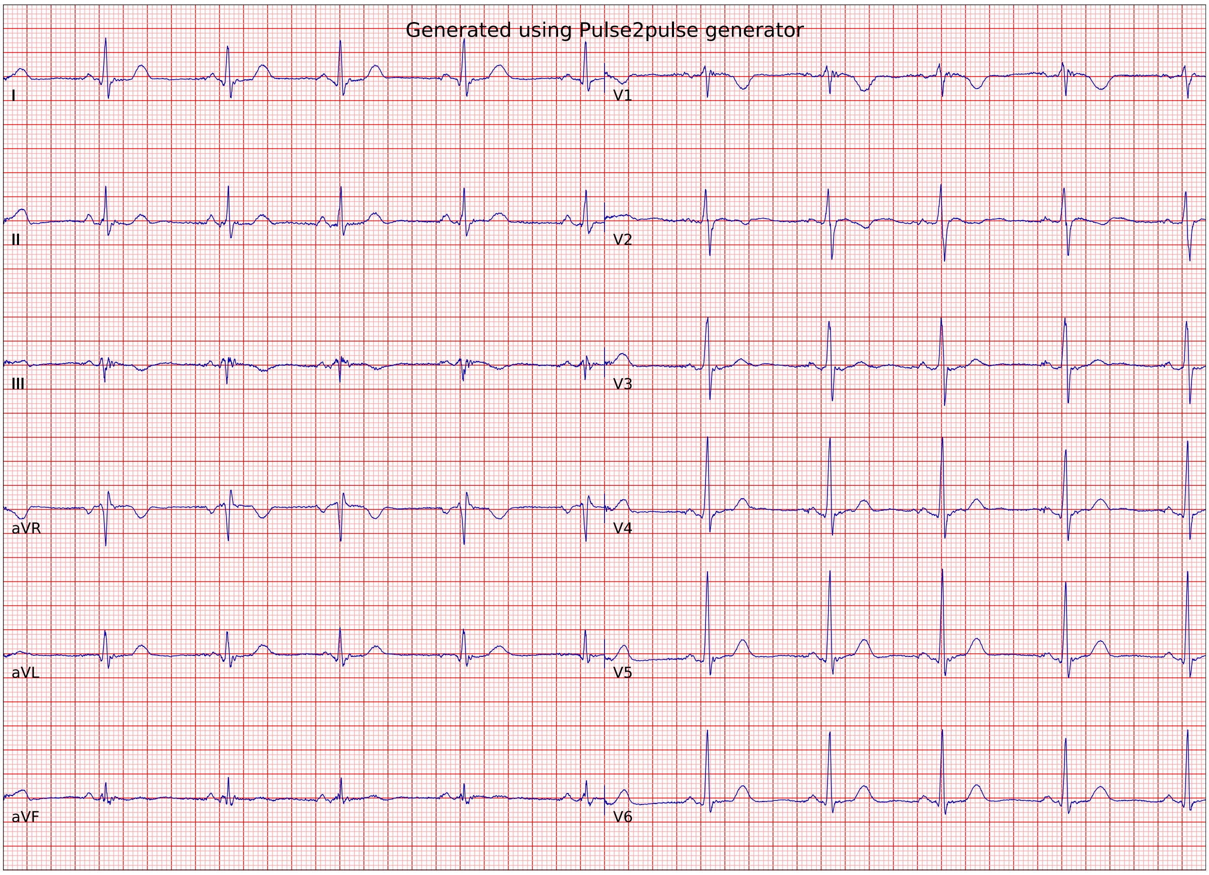
Generated using Pulse2pulse generator
I
V1
II
V2
III
V3
aVR
V4
aVL
V5
aVF
V6

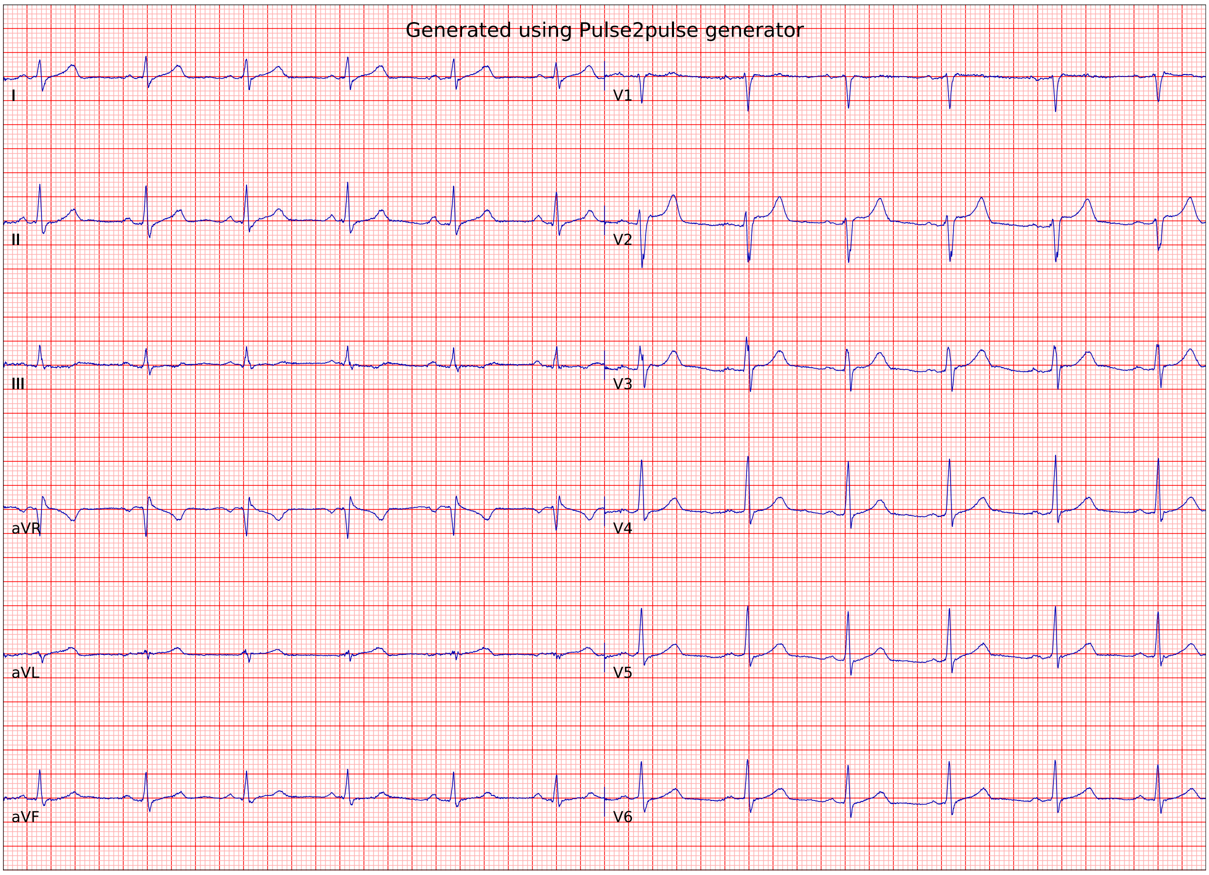
Generated using Pulse2pulse generator
I
V1
II
V2
III
V3
aVR
V4
aVL
V5
aVF
V6

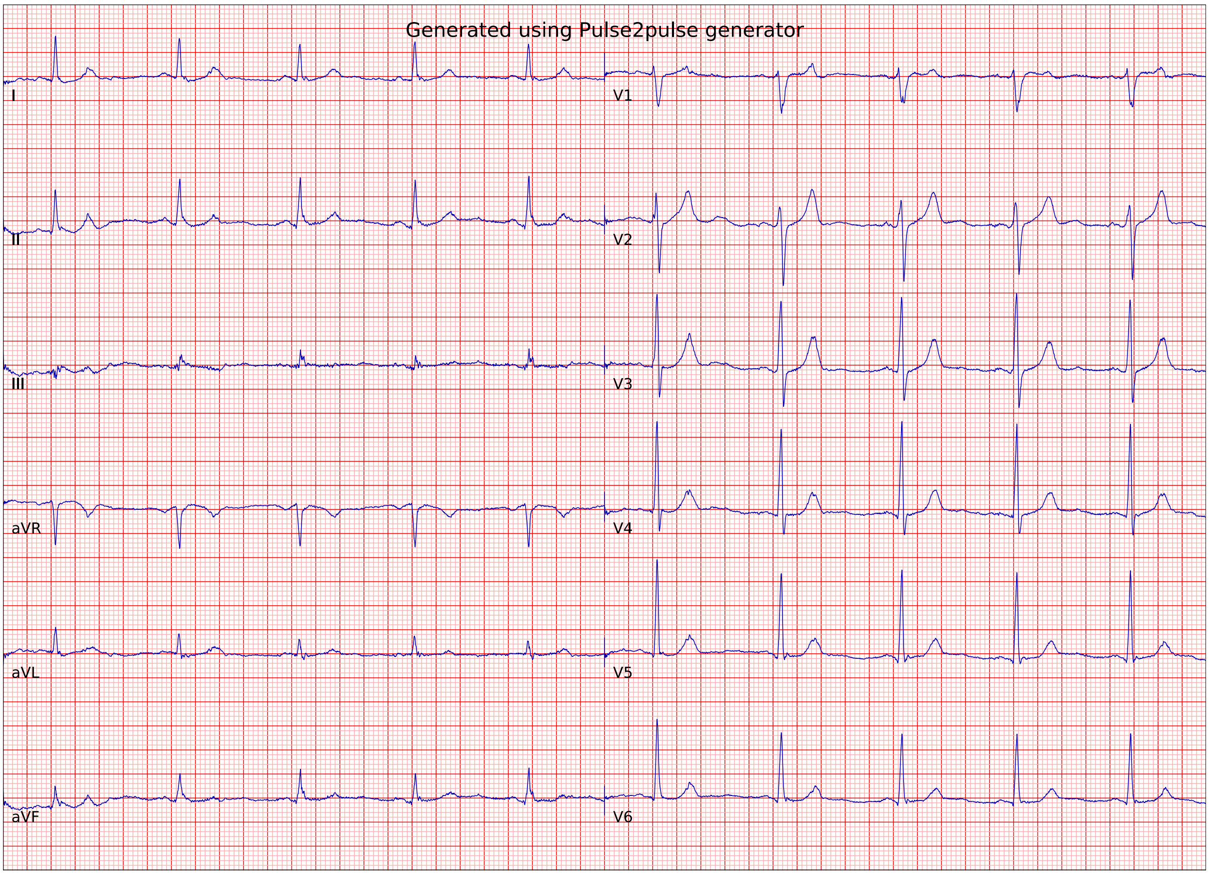
Generated using Pulse2pulse generator
I
V1
II
V2
III
V3
aVR
V4
aVL
V5
aVF
V6

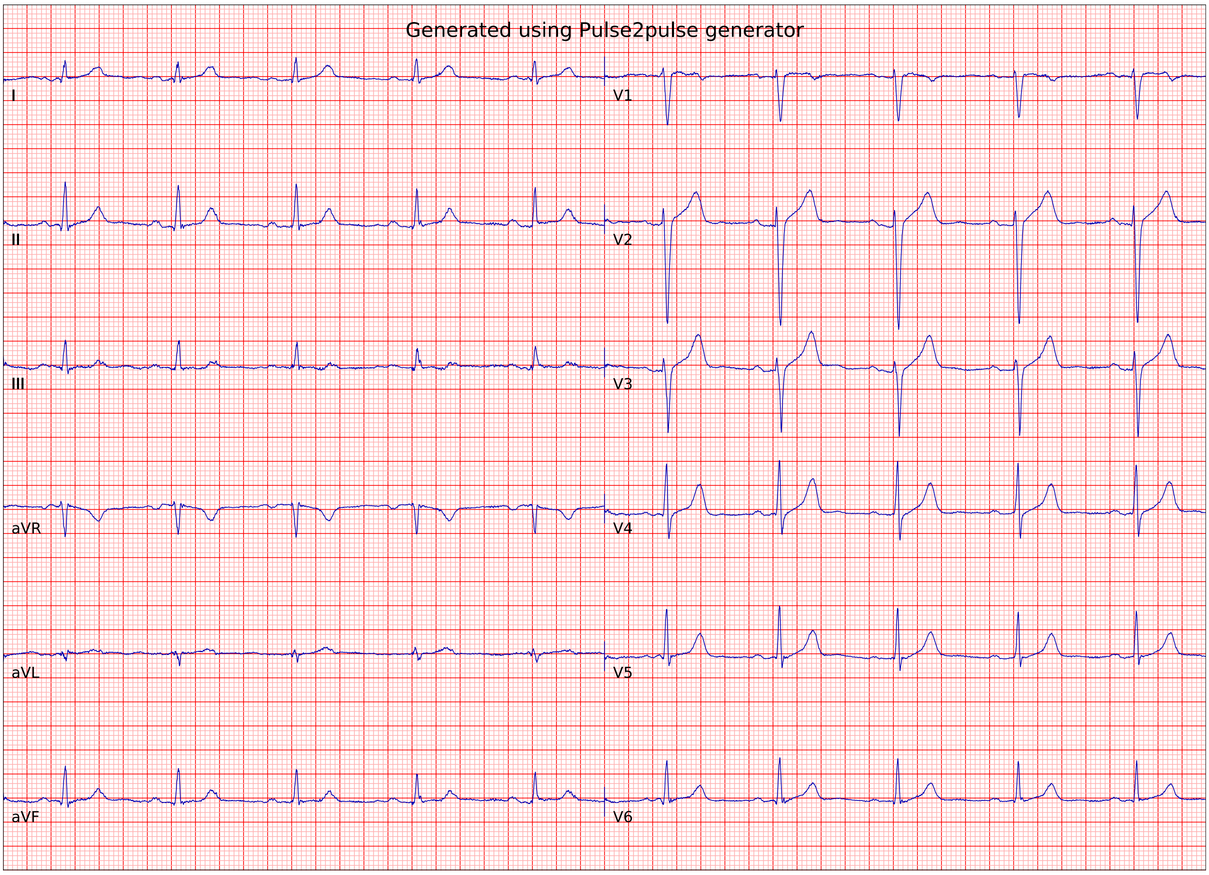
Generated using Pulse2pulse generator
I
V1
II
V2
III
V3
aVR
V4
aVL
V5
aVF
V6

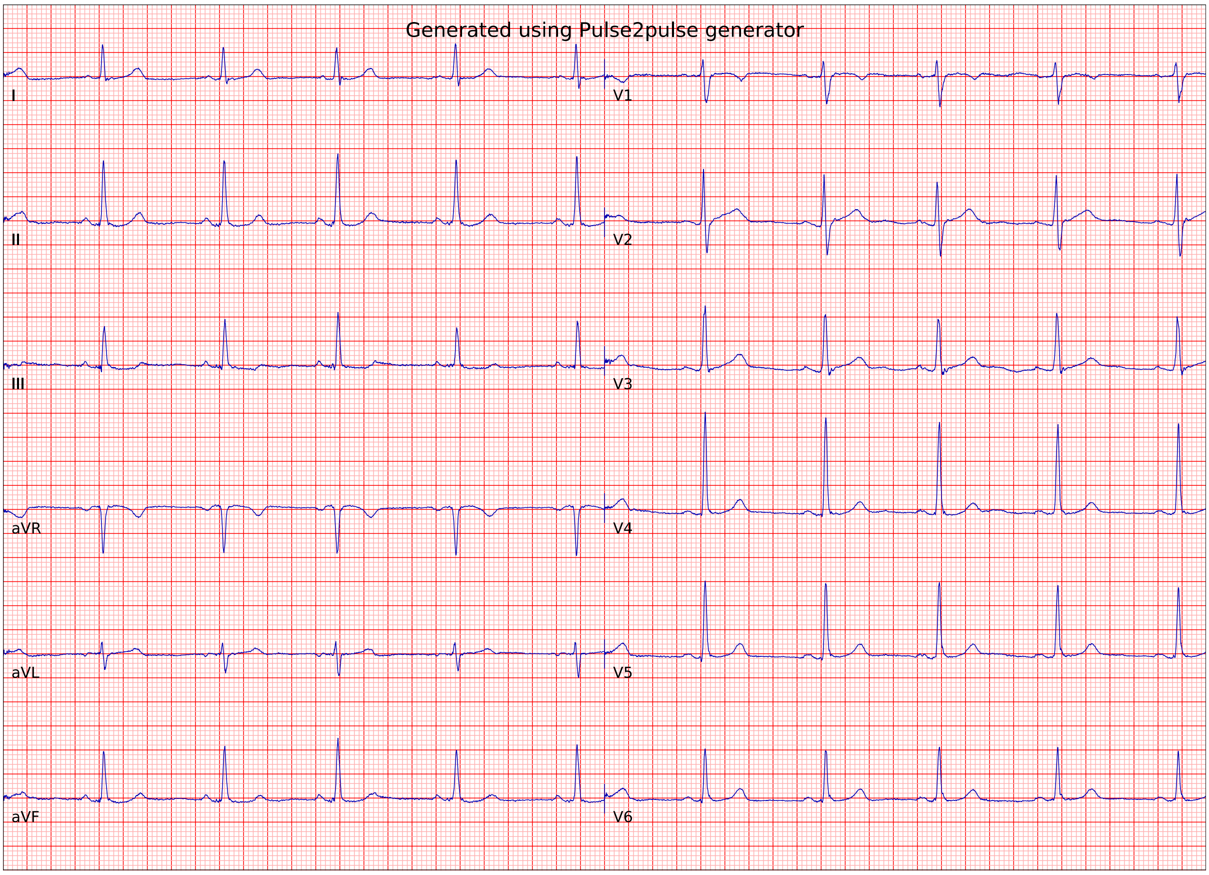
Generated using Pulse2pulse generator
I
II
III
aVR
aVL
aVF
V1
V2
V3
V4
V5
V6

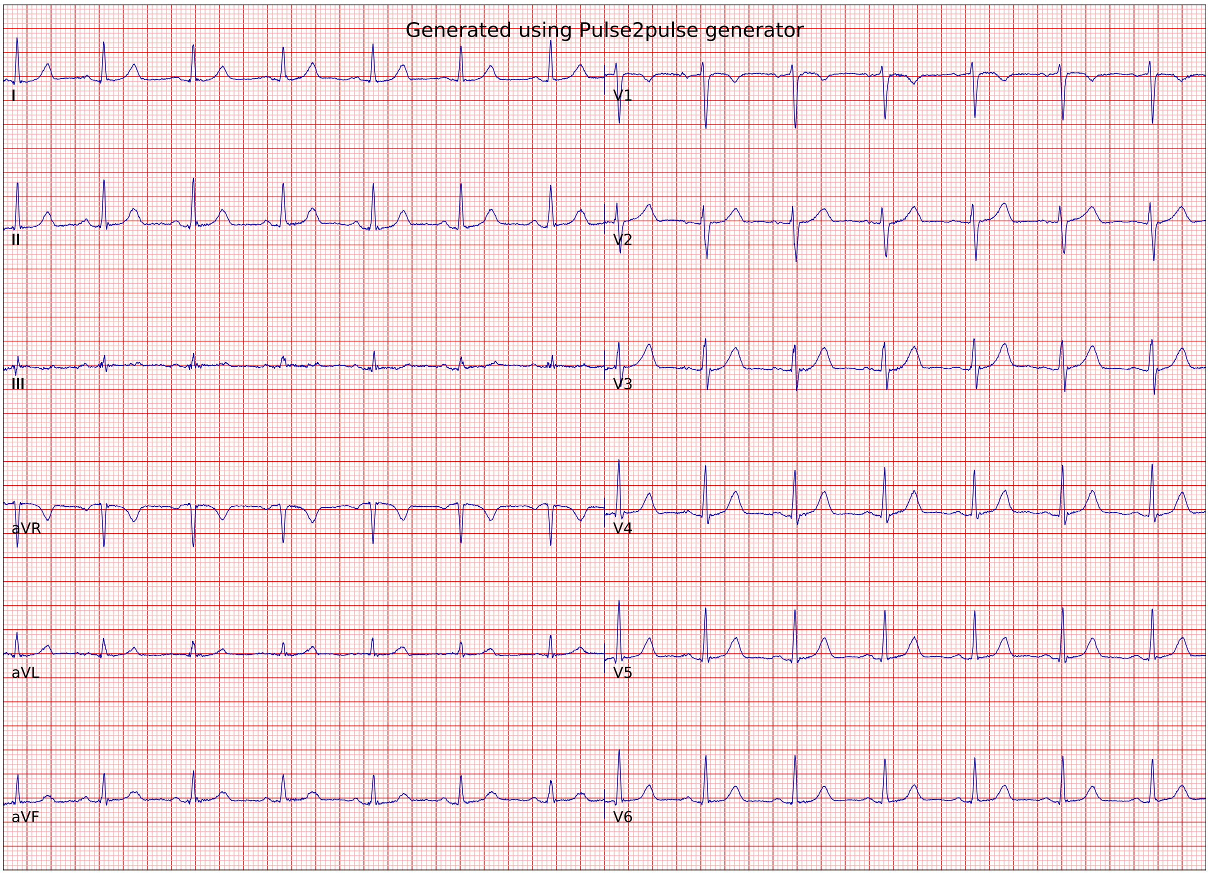
Generated using Pulse2pulse generator
I
II
III
aVR
aVL
aVF
V1
V2
V3
V4
V5
V6

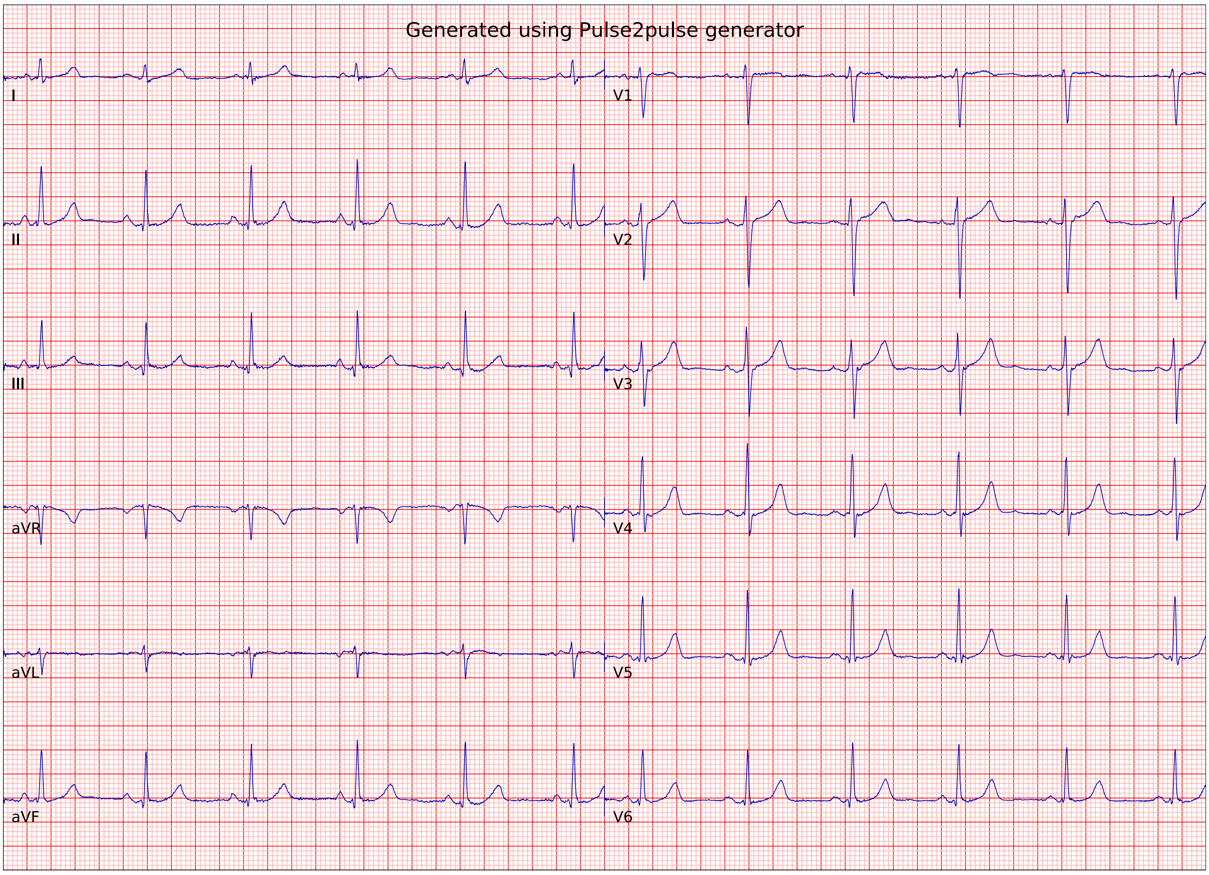
Generated using Pulse2pulse generator
I
V1
II
V2
III
V3
aVR
V4
aVL
V5
aVF
V6

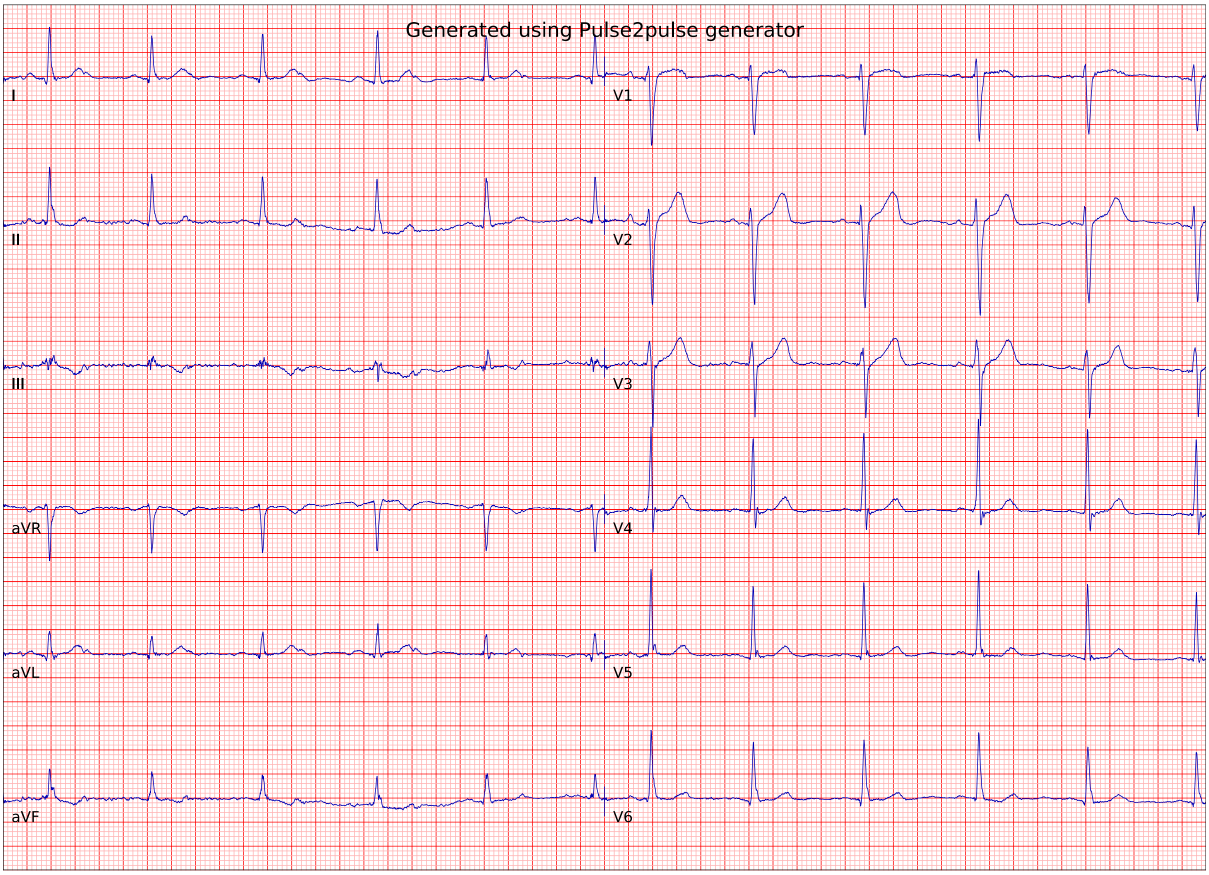
Generated using Pulse2pulse generator
I
V1
II
V2
III
V3
aVR
V4
aVL
V5
aVF
V6

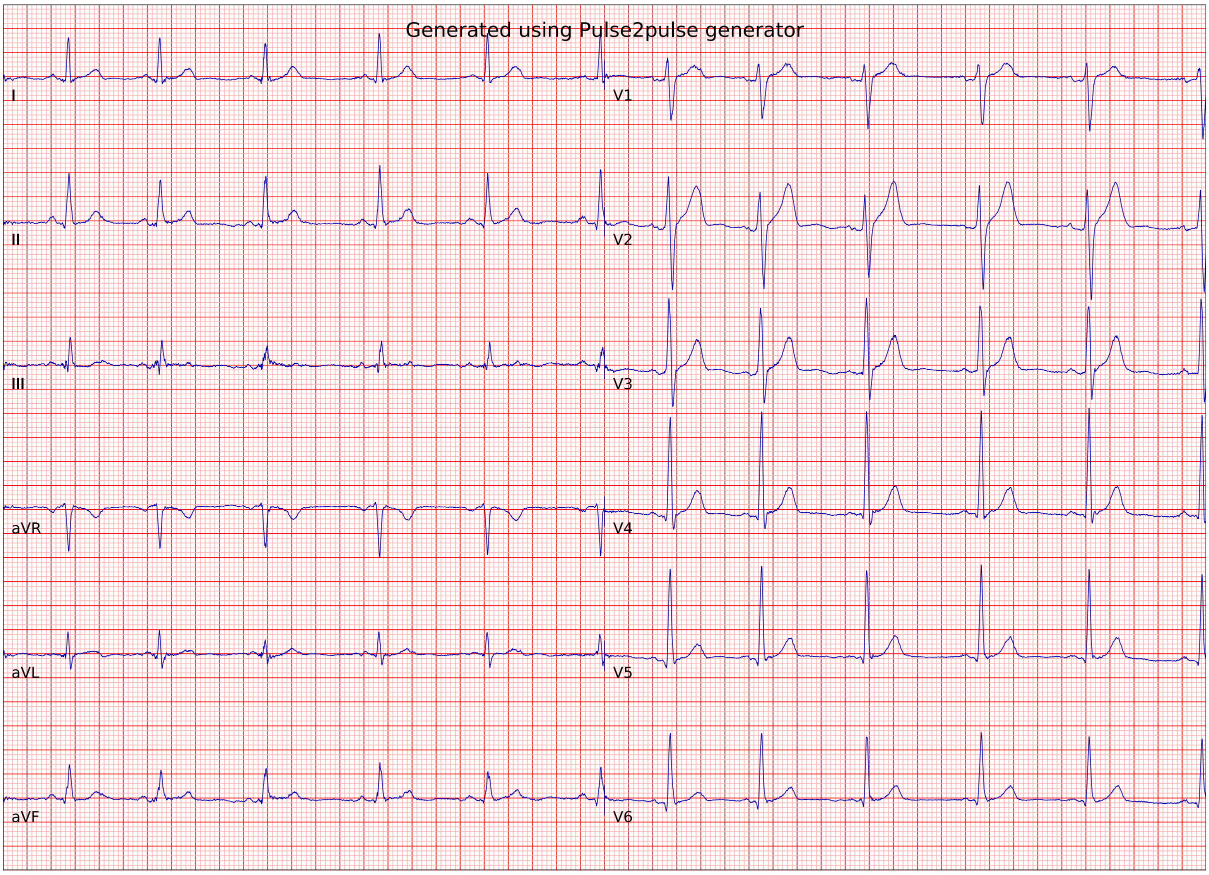
Generated using Pulse2pulse generator
I
II
III
aVR
aVL
aVF
V1
V2
V3
V4
V5
V6

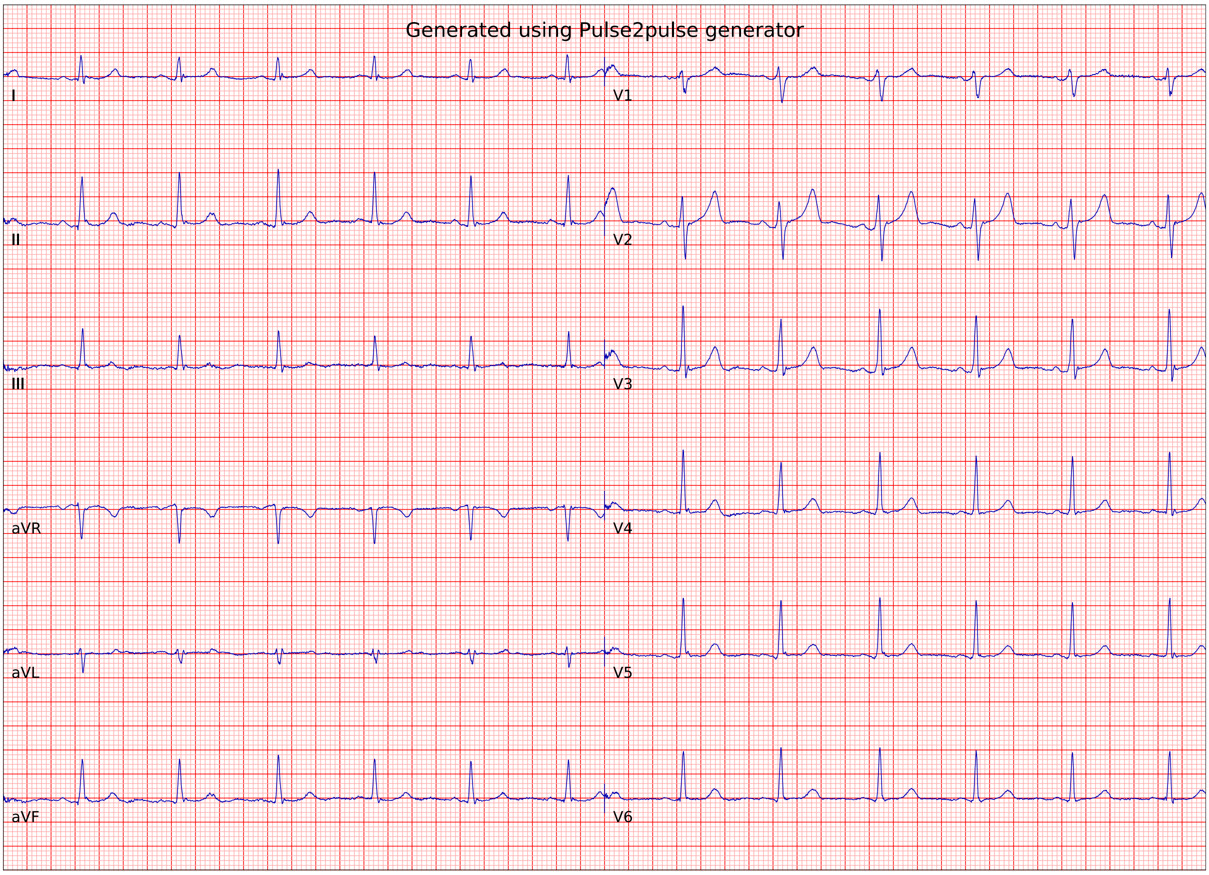
Generated using Pulse2pulse generator
I
II
III
aVR
aVL
aVF
V1
V2
V3
V4
V5
V6

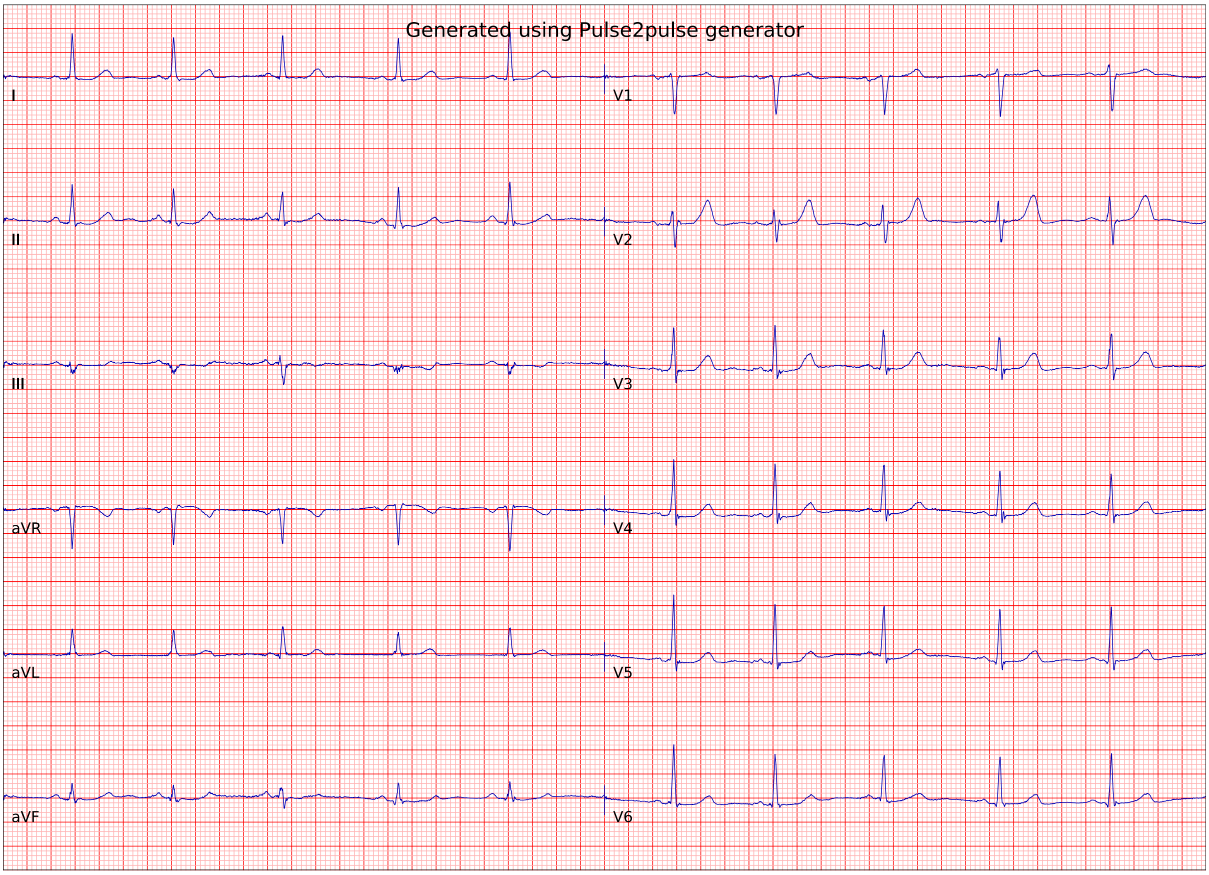
Generated using Pulse2pulse generator
I
II
III
aVR
aVL
aVF
V1
V2
V3
V4
V5
V6

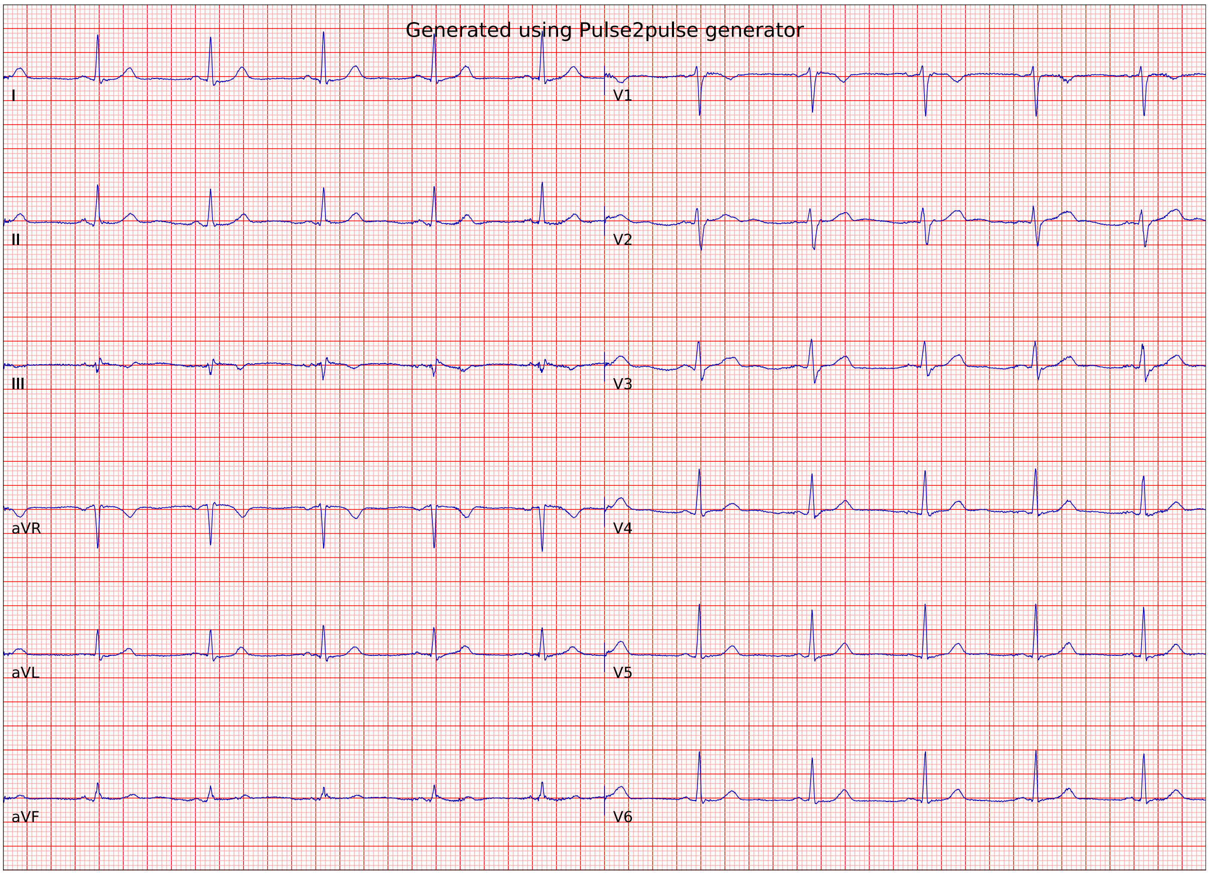
Generated using Pulse2pulse generator
I
II
III
aVR
aVL
aVF
V1
V2
V3
V4
V5
V6

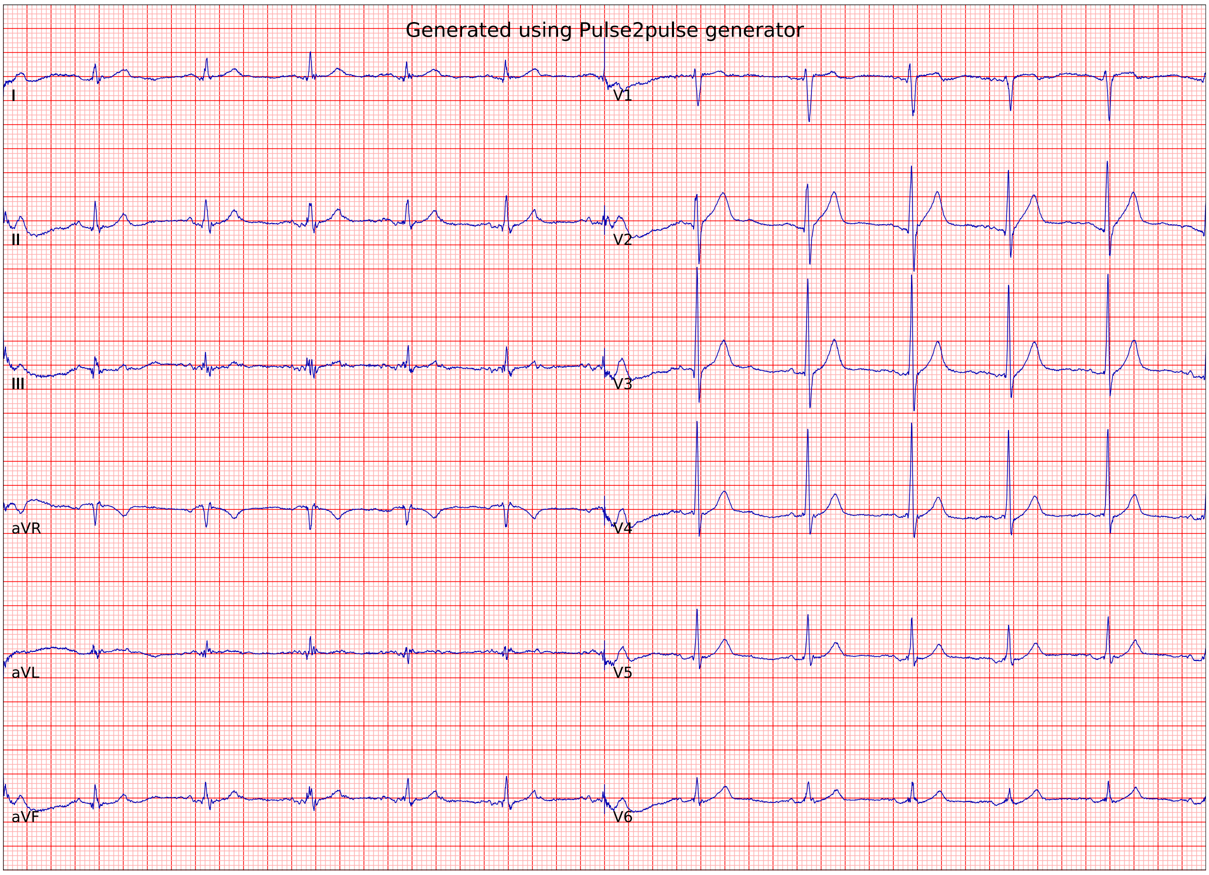
Generated using Pulse2pulse generator
I
II
III
aVR
aVL
aVF
V1
V2
V3
V4
V5
V6

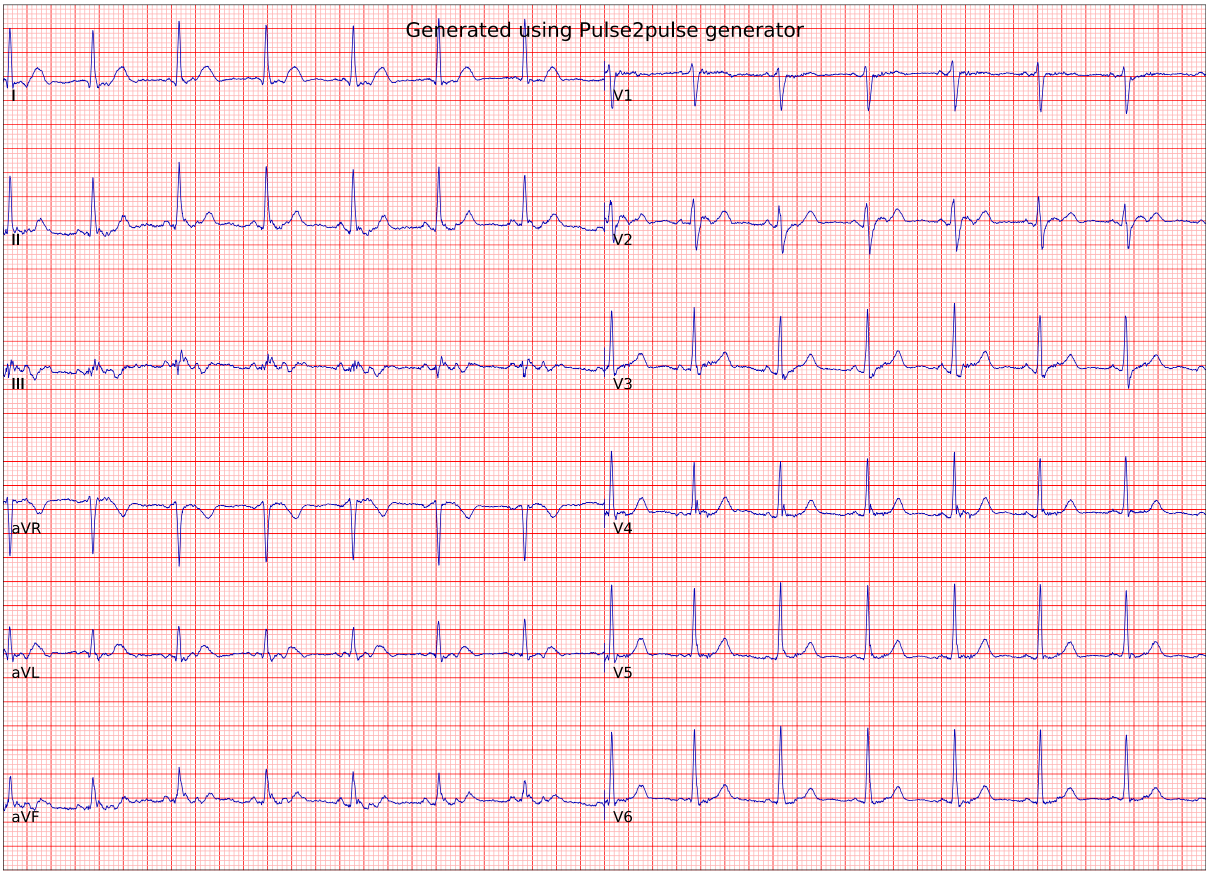
Generated using Pulse2pulse generator
I
II
III
aVR
aVL
aVF
V1
V2
V3
V4
V5
V6

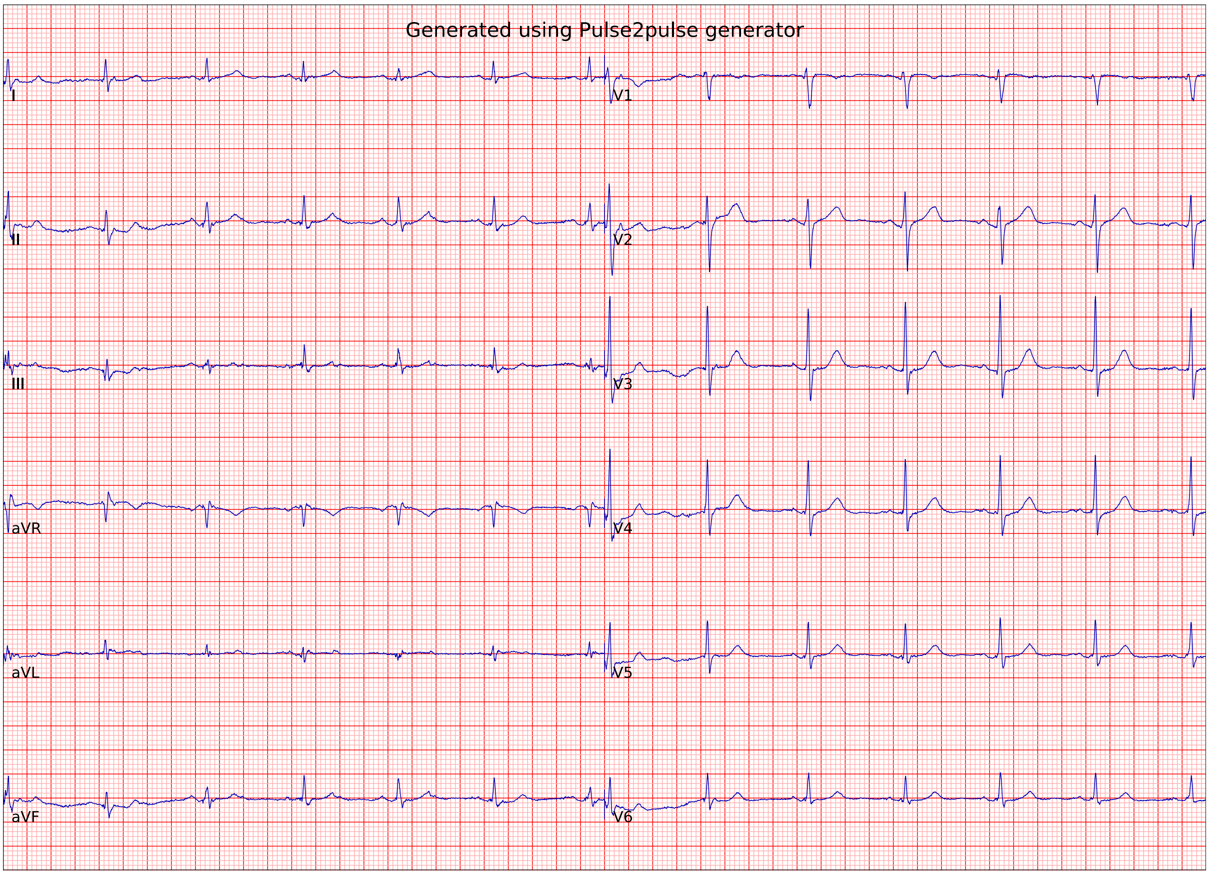
Generated using Pulse2pulse generator
I
II
III
aVR
aVL
aVF
V1
V2
V3
V4
V5
V6

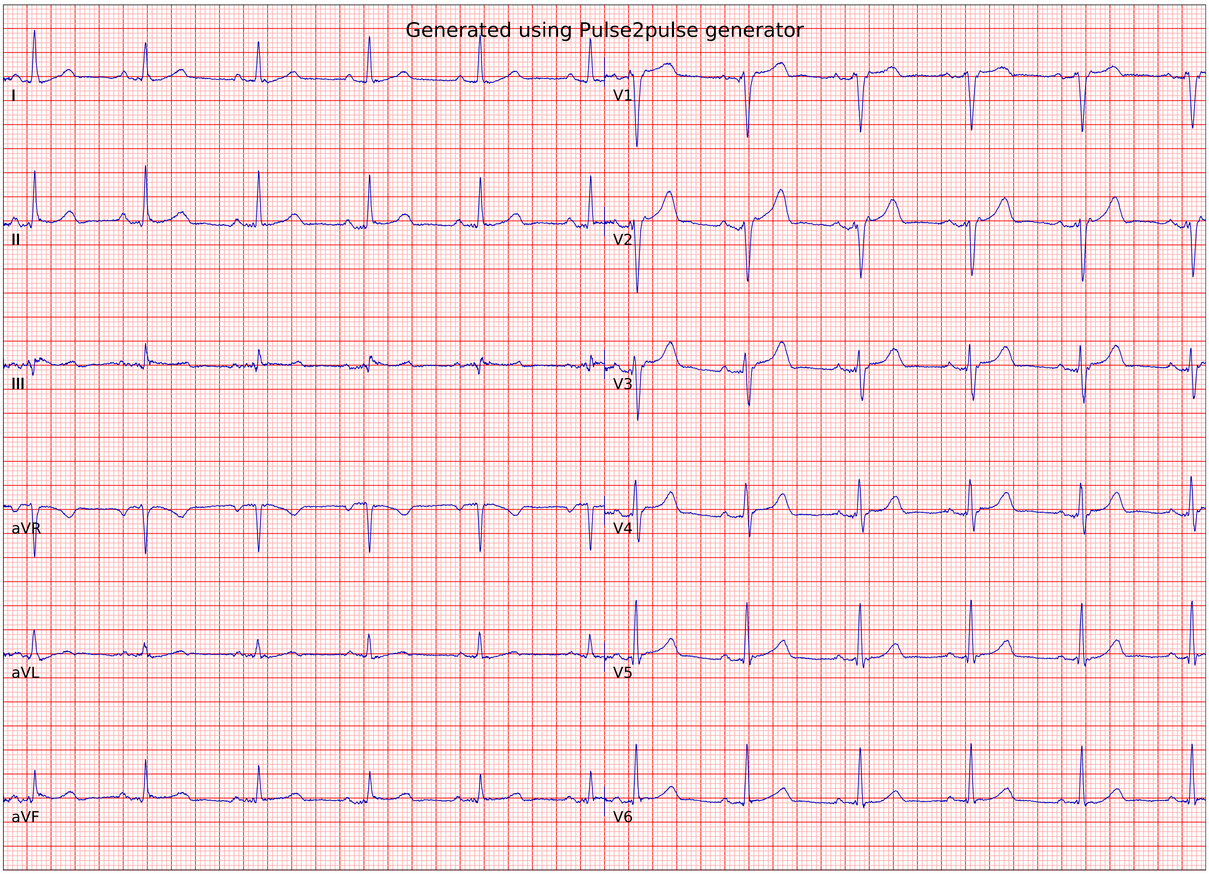
Generated using Pulse2pulse generator
I
II
III
aVR
aVL
aVF
V1
V2
V3
V4
V5
V6

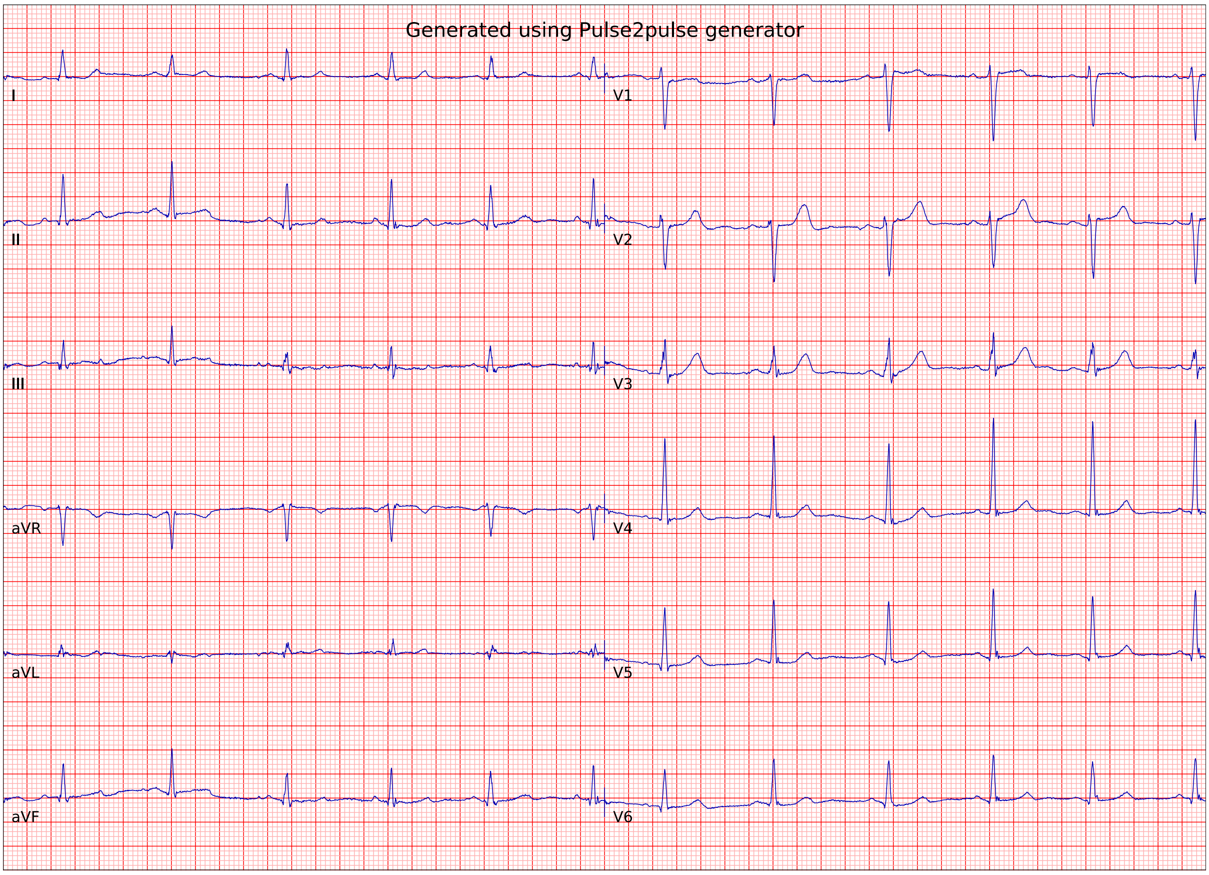
Generated using Pulse2pulse generator
I
II
III
aVR
aVL
aVF
V1
V2
V3
V4
V5
V6

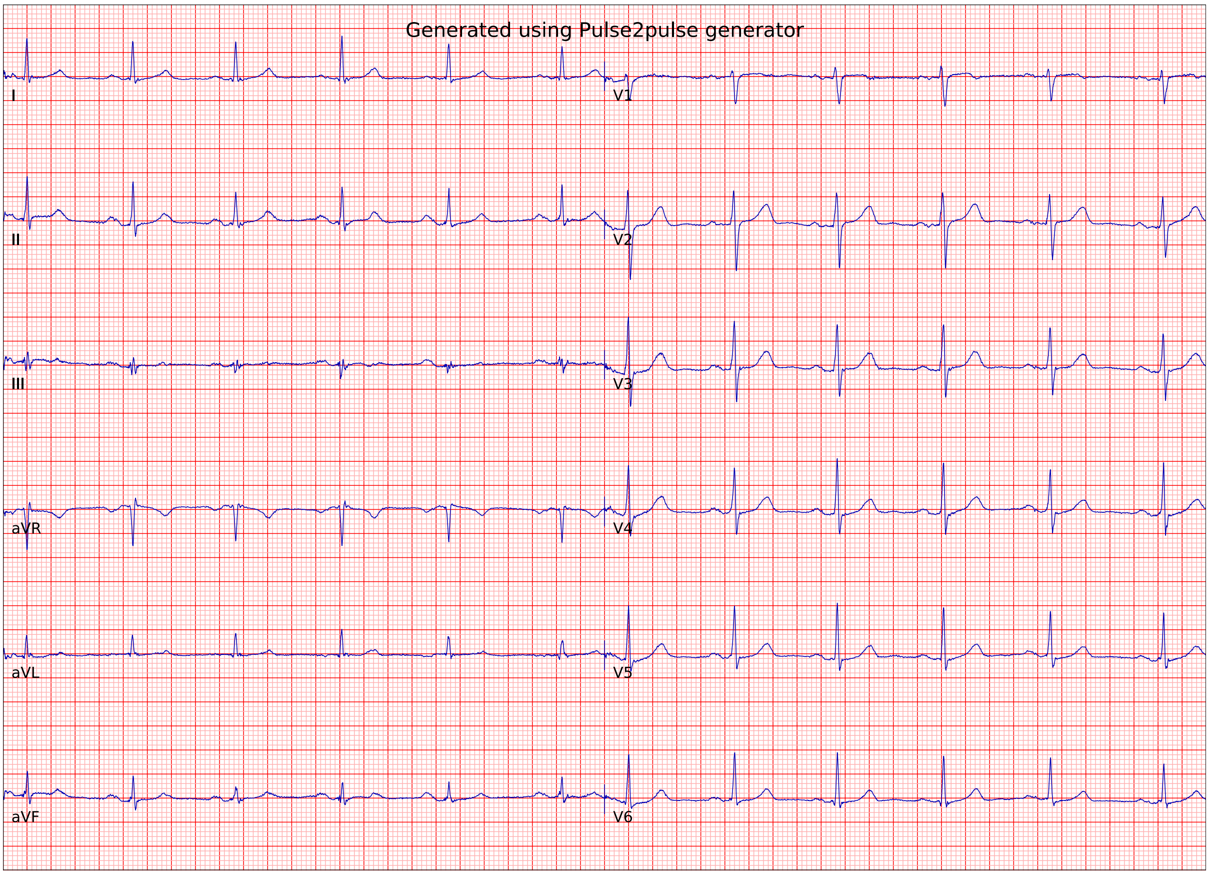
Generated using Pulse2pulse generator
I
II
III
aVR
aVL
aVF
V1
V2
V3
V4
V5
V6

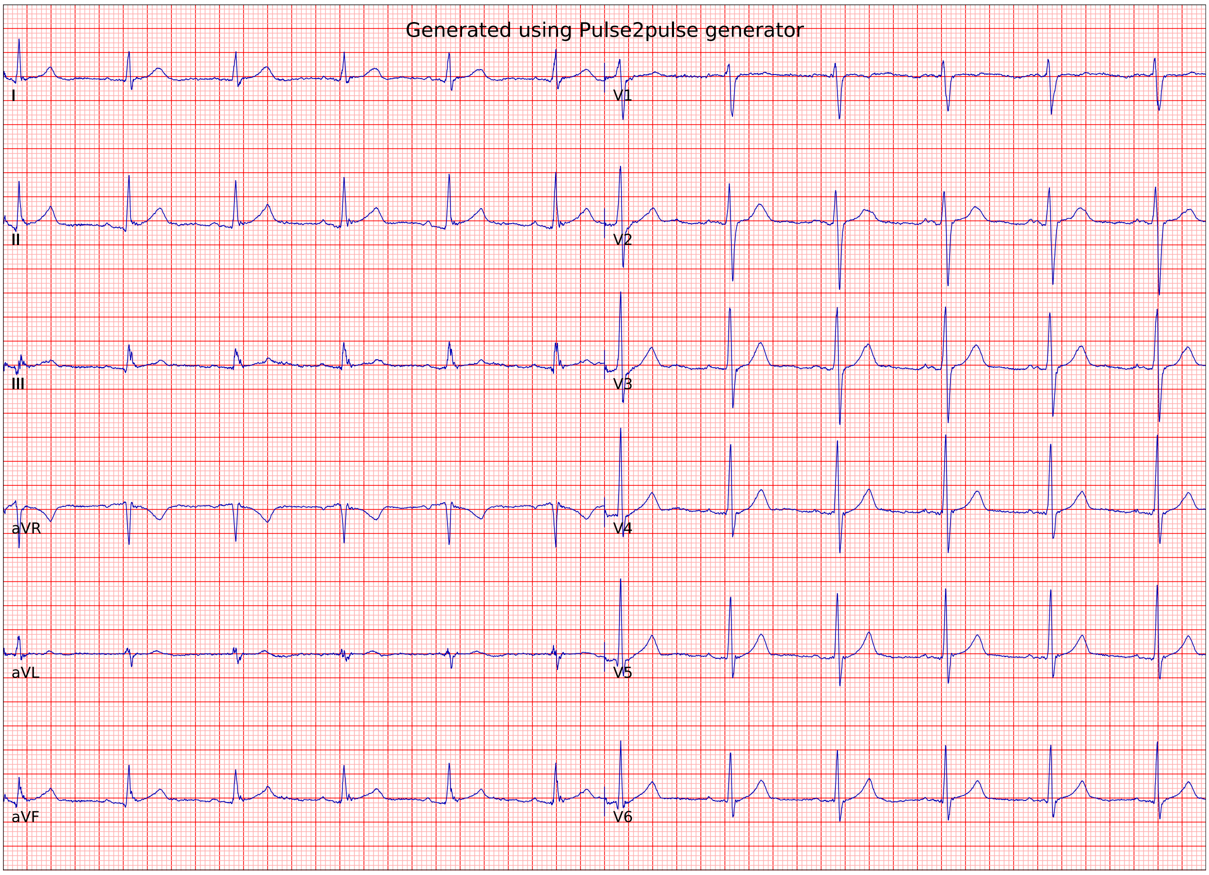
Generated using Pulse2pulse generator
I
II
III
aVR
aVL
aVF
V1
V2
V3
V4
V5
V6

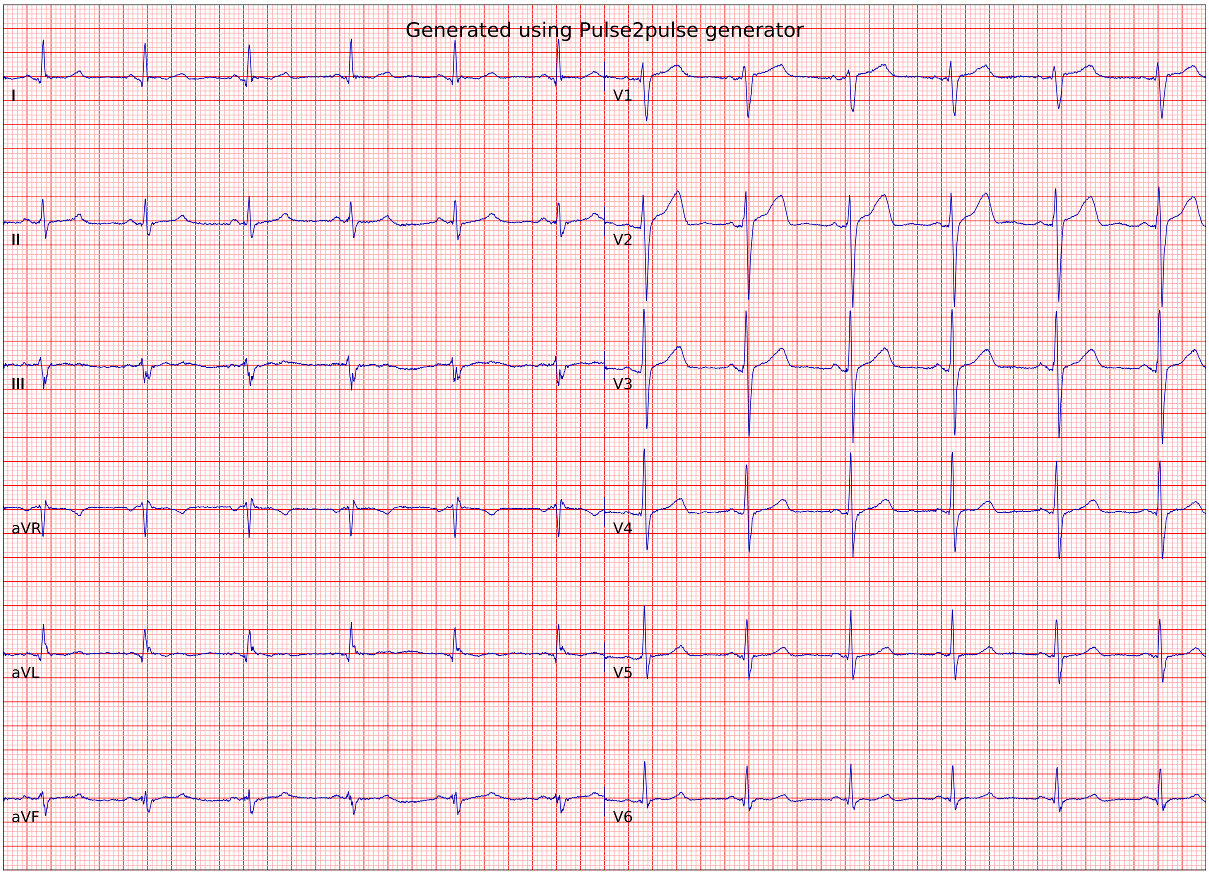
Generated using Pulse2pulse generator
I
II
III
aVR
aVL
aVF
V1
V2
V3
V4
V5
V6

Figure S1. Twenty randomly selected DeepFake ECGs. All the ECGs were selected from the Normal DeepFake ECGs.

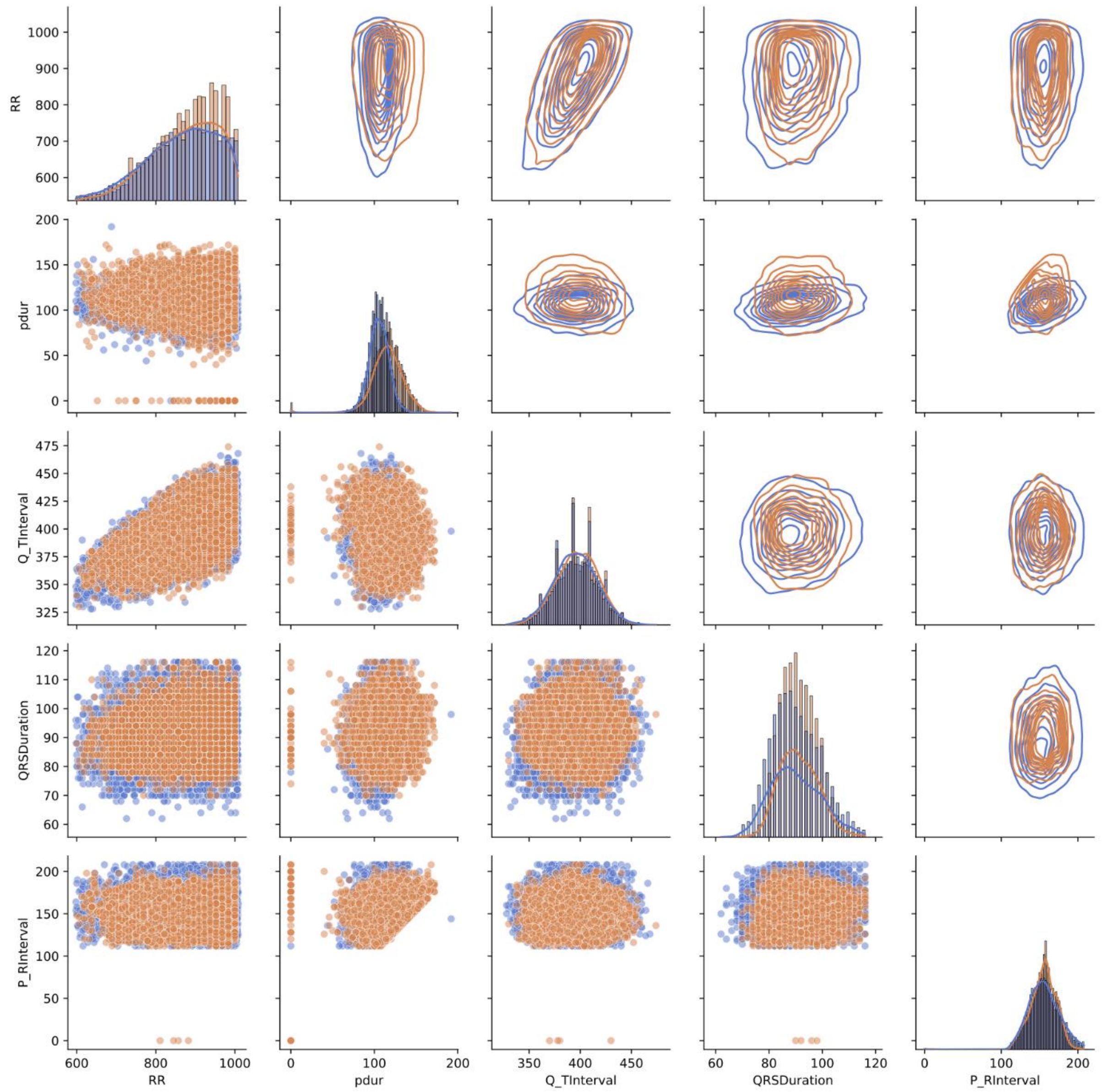

(a) From WaveGAN*

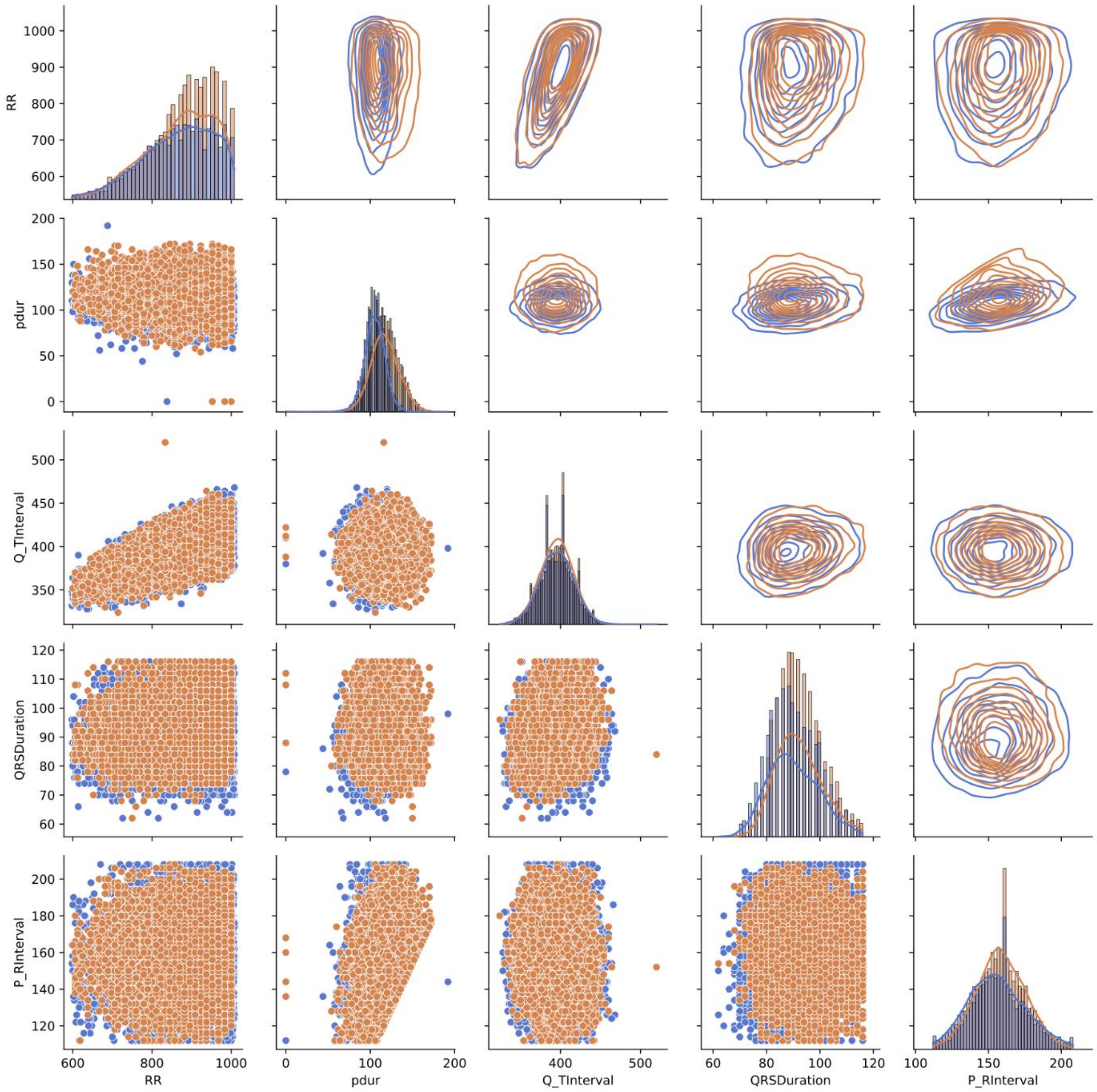

(b) From Pulse2Pulse

Figure S2. The comparisons of the real and DeepFake distributions. Blue color plots represent real normal ECG distributions. Orange color plots represent the distribution of fake ECGs generated by (a) WaveGAN* and (b) Pulse2Pulse.
